# Enhanced Ion Mobility in Helmholtz Layer Enabling Ultrathick Electrodes

**DOI:** 10.1021/acsnano.5c04343

**Published:** 2025-04-28

**Authors:** Yuanzhen Wang, Florian Aubermann, Joachim P. Spatz

**Affiliations:** † Max Planck Institute for Medical Research@Bildungscampus Heilbronn, Dept. of Cellular Biophysics, D-74076 Heilbronn, Germany; ‡ Institute for Molecular Systems Engineering and Advanced Materials, Heidelberg University, D-69120 Heidelberg, Germany; § Max Planck School Matter to Life@Bildungscampus Heilbronn, D-74076 Heilbronn, Germany

**Keywords:** microfluidic chip, ion diffusion, Helmholtz layer, 3-dimensional ultrafine copper fiber fleece/graphite composite, ultrathick electrode

## Abstract

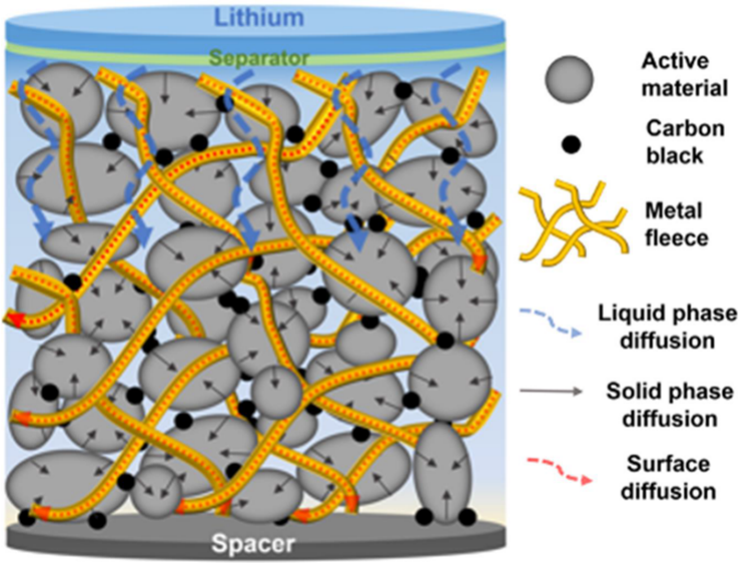

A previously unknown mechanism for enhancing the diffusivity of lithium ions in liquid electrolytes is reported by immersing a charged copper surface in an electrolyte, which forms a laterally ultramobile Helmholtz layer at the copper-electrolyte interface. A microfluidic chip in combination with Raman spectroscopy and molecular ab initio dynamic simulations allowed for a quantitative study and mechanistic description of the diffusion enhancement. The synthesis of an ultrafine copper fiber fleece with a large copper surface area enables the fabrication of a 3-dimensional graphite/copper fleece composite electrode. Such electrodes demonstrate enhanced ion diffusivity and electrical conductivity compared to traditional electrodes without copper fleece but with copper foil. This electrode design allows electrochemically functional ultrathick anodes of up to 1.2 mm thickness and 32 mAh/cm^2^ areal capacity in half-cell format. The fleece electrodes use half the amount of copper compared to conventional foil-based electrodes and therefore provide significantly higher volumetric and gravimetric energy densities.

## Introduction

Electrochemical properties of materials are strongly affected by the mobility of their charge carriers. For example, the performance of a battery electrode is strongly affected by charge carrier mobility. Sluggish lithium-ion (Li^+^-ion) transport kinetics and limited electrical conductivity in the active material of electrodes are critical bottlenecks for today’s lithium-ion battery (LiB) performance.^1−4^ Ion transport in electrodes occurs through tiny pores filled with the electrolyte. Ion mobility in liquid electrolytes can be enhanced by reducing the solvation shell of the ions. For example, Pan et al.^5^ systematically discussed the lithium solvation shell structures in carbonate electrolytes and pointed out that the solvation structure is crucial for rapid ion transport and fast charging performance of LiBs. Previous studies have also found that the ion diffusion process in liquid electrolytes is enhanced in the presence of a charged surface.^6−8^ Based on electric double layer (EDL) theory and computational calculations,^9−11^ an excess charge of a metallic surface breaks the complex between ions and its solvation shell, e.g., in the case of Li^+^-ions, EC and DMC molecules are separated from the ion. Instead, ion-electron pairs are established on the metal surface, thereby enabling a much higher mobility compared with the cation–anion pairs or aggregates within the bulk electrolyte. So far, literature only provides qualitative estimations or theoretical calculations and simulations of ion mobility along charged surfaces.^6−8^ To the best of our knowledge, this paper reports for the first time quantitative experimental studies of ion diffusion enhancement in an electrolyte by a charged metal surface.

Many studies on batteries addressed Li^+^-ion diffusivity in active materials by material modifications from molecular-^12,13^ to mesoscale morphology optimization.^14,15^ In today’s standard Li^+^-ion batteries, ion diffusion occurs predominantly through liquid electrolytes under the influence of an electric field and is in the range of approximately 10^–6^ cm^2^/s. The Li^+^-ion flux in electrodes is strongly influenced by the electrolyte, the porosity of the active material, which is filled with the electrolyte, the tortuosity of the pore structure, and temperature.^16^ Since Li^+^-ion diffusion through active materials in today’s standard electrodes is limited, this has immediate consequences for the overall design of batteries. Electrode thickness has to be limited to get ions from one side to the other side of an electrode on reasonable time scales. Therefore, electrodes in battery cells do not exceed a thickness of ca. 100 μm (single-sided coating)^17^ and have to be stacked to sum up to reasonable energy capacities. In recent studies, thick cathodes based on layered Ni-rich and olivine LiFePO_4_ cathodes can reach ∼200 μm thickness with dry electrode technology.^18,19^

The quantitative determination of ion diffusivity values within electrodes is complex. In a typical Li^+^-ion graphite anode, the Li^+^-ion diffusion values reported differ, ranging from 10^–5^ to 10^–12^ cm^2^/s.^1,6,20−22^ These large variations of diffusion values indicate that in the multiphase compound of an electrode, there are several simultaneous transport mechanisms for Li^+^-ions. From a nano- to a macroscopic perspective, the diffusion pathways of Li^+^-ions within a graphitic carbon electrode are as follows: starting at the separator, Li^+^-ions undergo (i) liquid (electrolyte)-phase diffusion within the porous electrode, (ii) solid-phase diffusion into the interlayer of graphite particles, and (iii) solid-phase diffusion within the crystal structure of the graphite until they finally accomplish intercalation.^23^ Different approaches are reported in the literature to separate and quantify the individual diffusion processes.^21,24,25,22,26−28^ Diffusivity values obtained by electrochemical impedance spectroscopy (EIS) are known to be very sensitive to differences in the experimental setup, electrode heterogeneity, and solid electrolyte interface (SEI) resistance. Therefore, EIS is usually only used for relative comparisons between different electrodes.^24,29,30^ Galvanostatic intermittent titration technique (GITT) is applied to measure the effective diffusion rate of lithium into graphite particles (above-described solid-phase diffusion processes (ii) and (iii)^22,25,26^). These rates are directly affected by the local Li^+^-ion concentration presented to the graphite particles after liquid-phase diffusion (process (i)), which in turn is affected by the length of the diffusion path of ions through the electrode materials, i.e., the thickness of the electrode. Therefore, GITT provides solid-state diffusion values which are affected by electrode thickness and therefore reflect the overall diffusivity of ions through the macroscopic electrode.^1^

## Results and Discussion

**Figure 1:**
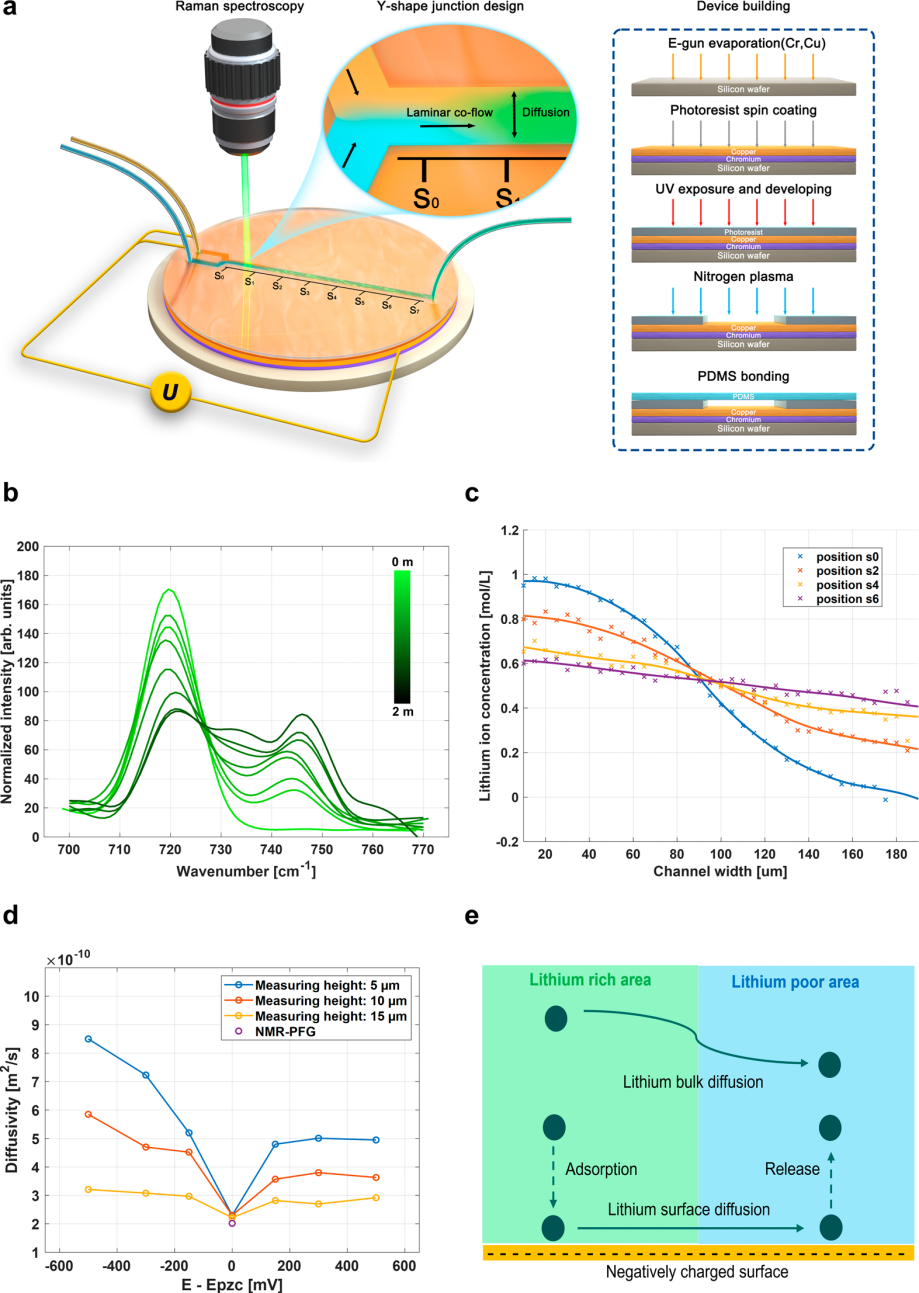
(a) Experimental setup illustrating the working principle of the diffusion process in a Y-shaped microfluidic channel and the device design; (b) Raman spectroscopy of a Li^+^-ion complex formation with EC ligands which impact the O–C–O bonding vibration, thereby causing Raman peak shifting from 720 to 733 cm^–1^ at different concentrations; simultaneously P–F stretching is observed at 744 cm^–1^ when more salt is dissolved; (c) Raman-visualized Li^+^-ion gradient across the channel at different sampling points (S0 to S7) at 20 μm objective lens height and 0 V against zero charge potential; the ion diffusion process is perpendicular to the laminar coflow; (d) Li^+^-ion diffusivity calculated by Fick’s law measured at different channel heights and different copper surface potentials; the diffusivity increases with the offset potential and also changes with the objective lens height settings during Raman measurements; and (e) scheme of the enhanced ion diffusivity along the charged copper surface which dominates the overall diffusion process within the microfluidic channel.

To quantify ion diffusion in an electrolyte supported by an immersed charged metal surface, we developed a novel microfluidic experiment combined with Raman spectroscopy (details are given in Supporting Information I). Figure 1a illustrates the design of the electrochemical setup. A 5 nm thick adhesive layer of chromium is deposited onto a Si-wafer, followed by a 100 nm thick layer of copper. To assemble the microfluidic Y-shaped channel, a photoresist is applied on the copper surface and selectively removed to form the walls of the microfluidic channel. A thin layer of PDMS is bonded onto the photoresist to seal the channel. The Li^+^-ion diffusion experiment is based on two laminar coflows with different Li^+^-ion concentrations presented in the microchannels, as described, e.g. by Peters et al.^31,32^ Two electrically grounded laminar flows of electrolytes, one with and one without Li^+^-ions, are injected into the two arms of the Y-shaped channel. As the flows reach the Y-junction, the different liquids get in contact with one another and enable equilibration by a lateral Li^+^-ion flow. However, due to the laminar flow characteristics, the two fluids do not mix immediately. Exchange between the two laminar flows is expected to occur slowly, i.e. at a greater distance from the Y-junction. Confocal Raman Spectroscopy is applied to spatially and temporally quantitatively measure the Li^+^-ion concentration perpendicular to the flow direction at different sites of the microfluidic channel.

In the electrolyte (1 M LiPF_6_ in 50:50 ethylene carbonate (EC): dimethyl carbonate (DMC)), the dissolved Li^+^-ions associate with solvent molecules and form solvent-separated ion pairs (SSIPs), contact ion pairs (CIPs), and aggregates. The ion–solvent association is mediated by ion-dipole interactions which impact the solvent molecule bond vibration frequencies, thus leaving a trace in the solvent molecules’ Raman spectrum. Specifically, we focus on the Li^+^-ion and EC ion–solvent association. A Li^+^-ion impacts the oxygen–carbon-oxygen bond (O–C–O) vibrational frequency of EC at 720 cm^–1^ by shifting it toward 733 cm^–1^ (Figure 1b).^33^ Meanwhile, P–F stretching is observed at 744 cm^–1^, indicating an increased amount of anions (PF_6_^–^).^34,35^ By eliminating fluorescence noise, normalizing, and comparing the intensity of the original O–C–O peak and the P–F peak, Li^+^-ion concentration is visualized spatially resolved and in real-time (the applied algorithm is described in Supporting Information II). Figure S6 presents the calibration of the normalized Raman spectrum ratio as a function of the LiPF_6_ concentration.

The Li^+^-ion concentration gradient from the lithium-rich to lithium-poor phase is extracted from Raman spectroscopy line scans along the channel cross-section width (perpendicular to the flow direction, Figure 1c). The concentration gradients at different sites of the microfluidic channel–position S0 to S7 with each position separated from the neighboring one by 10 mm along the direction of flow–are quantified at different time points.

When the two laminar flows first come in contact at the Y-junction (position S_0_), a clear concentration interface is established (Figure S7). The total width of the channel behind the Y-junction is 200 μm. At first, the voltage is set to 0 V against zero charge potential. At the channel entry (position S0), the concentration is 1 mol/L in one channel and 0 mol/L in the other channel. Measurements reveal that the concentration difference equilibrates once the Y-junction has been passed by the flow with increasing distance to the channel entries. At position S6, the concentration difference is 0.6 to 0.4 mol/L from one side to the other side of the channel’s cross-section. Based on Fick’s second law, the lithium-ion diffusion coefficient perpendicular to the flow direction is calculated to be 2.21 × 10^–10^ m^2^/s (eq S9). At 0 V against zero charge potential, this diffusion constant is independent of the channel height at which the spectra are taken (Figure 1c), and surface diffusion does not contribute to the bulk diffusion. The measured diffusivity value is also similar to results obtained by PFG-NMR and the essential diffusivity value of a bulk electrolyte of 2.02 × 10^–10^ m^2^/s.^16^

In contrast, the diffusion rate vertical to the flow direction increases substantially when an offset voltage is applied to the copper surface, regardless of whether the metal surface is positively or negatively charged (Figure 1d). Moreover, when the surface is negatively charged, the Li^+^-ion diffusivity is even greater than in the case of a positively charged surface. Measured diffusivity values also strongly depend on the height setting of the Raman microscope objective lens during spectra measurements as well as the applied voltage. When the measuring plane is closer to the charged surface, a higher diffusion rate along the channel cross-section is observed. At −500 mV against zero charge voltage, the effective diffusion rate within the channel measured at 15 μm height from the copper foil is enhanced by a factor of 1.5 in comparison to 0 mV. It increases to approximately 2-fold at 10 μm and 3-fold at 5 μm. This indicates that near the charged copper surface Li^+^-ion surface diffusion dominates strongly over bulk diffusion. In the case of a positively charged surface, the Li^+^-ion diffusion rate also increases. Here, the anion mobility is enhanced due to electroneutrality in the bulk electrolyte. There is a saturation of the diffusion coefficient observed at about +320 mV, since the enhancement of the diffusion process is limited by the essential mobility of Li^+^-ion in the bulk electrolyte. Importantly, the increase in electrical-potential-related diffusion suggests that the formation of an electric charge at the surface causes faster diffusion. Figure 1e illustrates the diffusion process within the microfluidic channel. Ions accumulate at the charged surface, and an ion flow from lithium-rich to lithium-poor domains is followed by the release of Li^+^-ions to the bulk. Figure S8 illustrates the positively charged situation.

Based on EDL theory and computational calculations,^9−11^ the copper surface excess charge breaks the complex between the Li^+^-ions, EC and DMC molecules. Instead, ion-electron pairs are established on the metal surface, thereby enabling a much higher mobility compared to the cation–anion CIPs, SSIPs, or aggregates within the bulk electrolyte. As a higher polarized potential is applied at the surface, more ions accumulate at the surface and are ‘activated’ for higher mobility, accelerating the mixing process vertically to the microfluidic flow direction within the channel.

**Figure 2:**
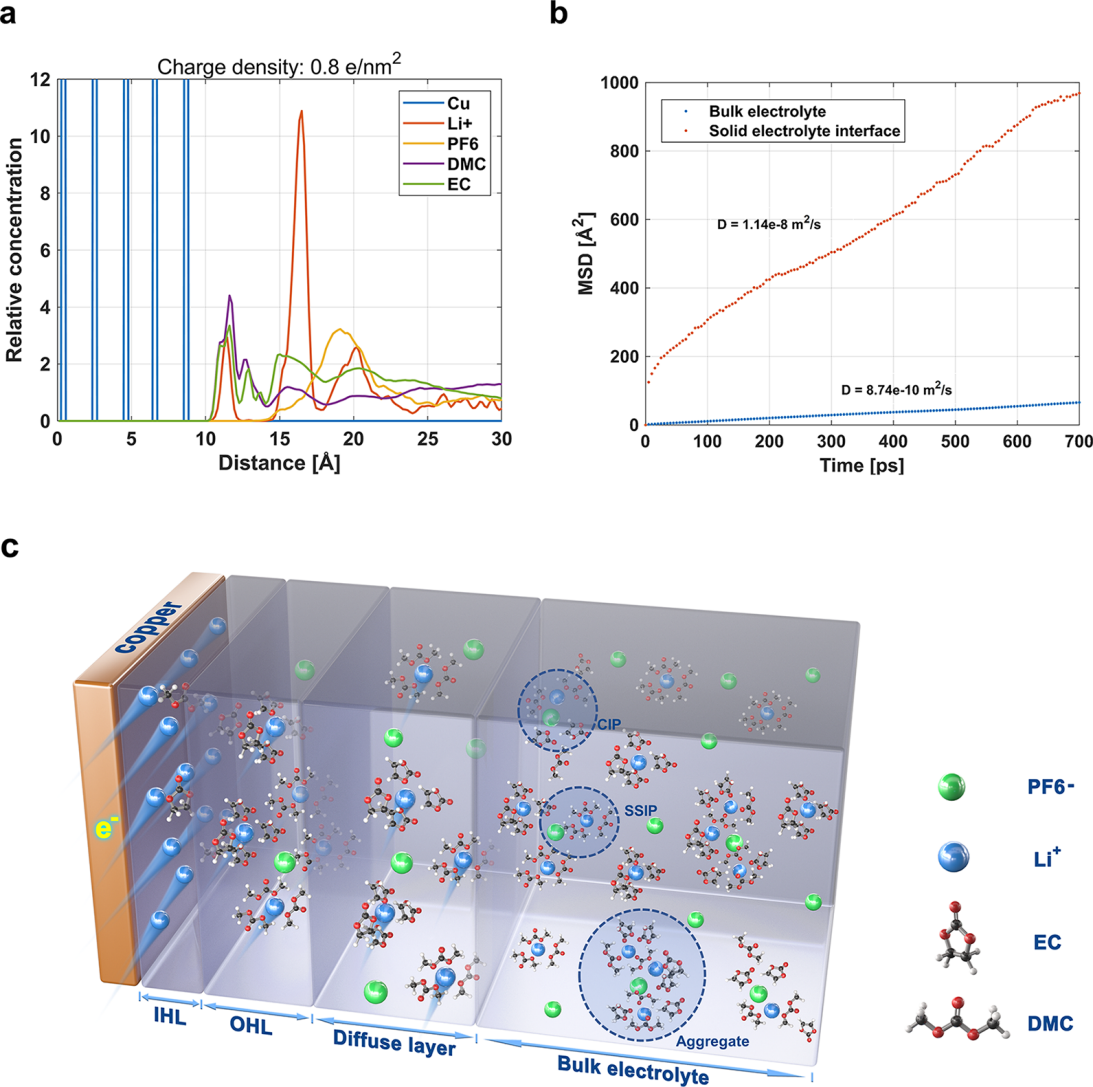
(a) MD simulation of the relative concentration near a charged surface (0.8 e/nm^2^); (b) diffusivity calculation based on the mean square displacement of ions over time. The diffusivity of ions on the charged copper surface (orange) and in the bulk (blue); (c) illustration of the MD simulation of the bulk electrolyte; solvent-separated ion pairs (SSIPs), contact ion pairs (CIPs), and large aggregates are generally formed in the bulk electrolyte and illustration of enhanced ion diffusion along a charged copper surface (electric double layer, EDL). EDL formation breaks the SSIPs, CIPs, and aggregates in the bulk electrolyte. Ions are able to form ion-electron pairs together with the excess charges within the inner Helmholtz layer (IHL) of the copper, enabling them to move ‘more freely’ without tracking the Li^+^-ion shell.

Full-scale ab initio molecular dynamic (MD) simulations (details shown in the Supporting Information III) are carried out based on the density functional theory (DFT). First, the bulk electrolyte is modeled, and a preset positive surface charge density is assigned to the copper surface. Due to the surface charge, the ions are redistributed along the surface, and the EDL is formed. Figure 2a shows the relative concentration profiles of the ions and solvent molecules. As the data indicates, two peaks of the cation concentration are observed near the surface, where the anion concentration, in contrast, is decreased. Here, the first Li^+^-ion peak indicates the position of the inner Helmholtz layer (IHL), while the second indicates the position of the outer Helmholtz layer (OHL). Within the EDL, the Li^+^-ion, EC and DMC are attached to the charged surface, while anions get repelled and vanish. Figure S11 presents the free EC, DMC, and Li^+^-ion coordinated EC/DMC, free Li^+^-ion, and free anions which are accumulated within the EDL and in bulk electrolyte. As the data show, a large portion of free Li^+^-ions is accumulated on the charged surface. In bulk electrolyte, all the Li^+^-ions are coordinated with solvent molecules and form SSIP, CIP, and aggregates. This indicates that the solvation shell or large aggregates of anions, solvent molecules, and cations are breaking up within the EDL due to the electrostatic force.

The diffusivity of Li^+^-ions is calculated based on the time evolution of its trajectory in the bulk electrolyte and on the surface (<18 Å); details are explained in Supporting Information III. As shown in Figure 2b, the ions at the surface exhibit a much higher mobility compared to that of the ions in the bulk. Linear fitting of mean square distance (MSD) and time reveals the diffusivity of surface ions to be 1.14 × 10^–8^ m^2^/s, which is about 56 times greater than Li^+^-ion diffusivity in the bulk electrolyte.

There is a difference between the diffusivity we calculated and the value given in the literature, presumably due to inaccurate force field calibration during simulation. Nevertheless, the ion and solvent molecule distribution reveals that in a liquid phase the ionic conductor, in this case the bulk electrolyte, as well as the anions and cations, always associate with the solvent molecule, resulting in the formation of a solvation shell or big aggregates that contain anions, cations, and solvent (Figure 2c). This phenomenon is well-described, both theoretically^36−38^ and experimentally.^39,40^ However, on a charged surface, such associations are deleted, causing ions and/or solvent molecules to directly interact with the charged surface via electrostatic forces.^41−43^ In an aqueous electrolyte, the cation solvation shell is robust.^38^ In contrast, in carbonate-based Li^+^-ion electrolytes, our MD simulations and the literature^9,44^ indicate that the Li^+^-ions are prone to desolvate and adsorb on the charged electrode surface in ‘bare’ form. Here, our experiments and calculations reveal that the breakage of the ion–ion and ion–solvent interactions at charged interfaces frees the ions from their ligand shells and thereby increases the mobility of the ions. As illustrated in Figure 2c, a Helmholtz layer (HL) is formed when the metallic surface is negatively charged. At the inner HL, neither ion–solvent nor ion–ion pairing exists anymore, and the large amounts of adsorbed ions form a compact HL at the surface. These bare ions interact with the excess electrons on the metallic surface and show ultrafast mobility. Also, the phenomenon is quite similar to the job-sharing diffusion at interfaces in solid-state mixed conductors described by Chen et al.^45^

**Figure 3:**
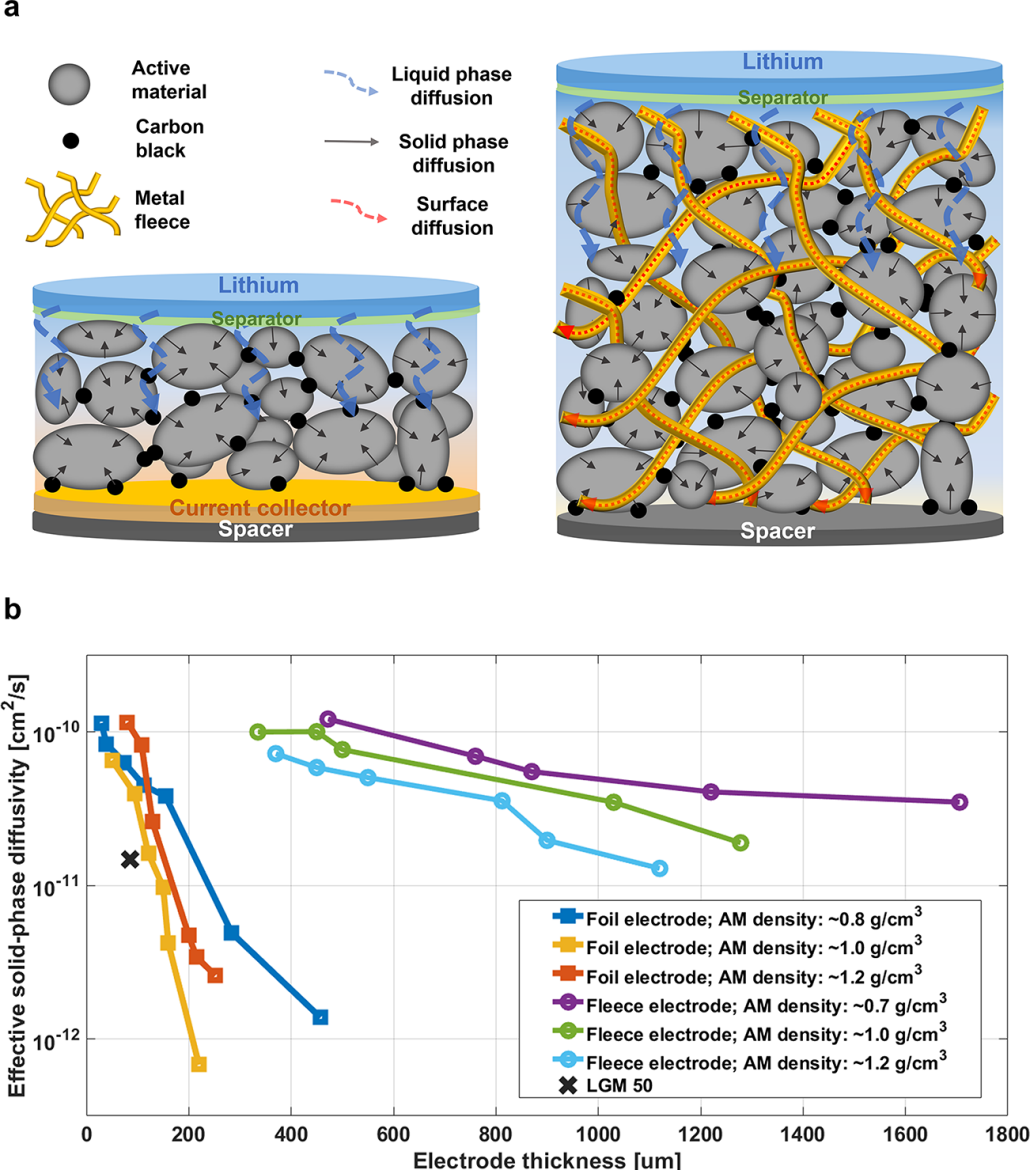
(a) Scheme depicting the structure of graphite-based anode half-cells: (left) solid and liquid-phase diffusion pathways in a copper foil-based anode; (right) solid phase diffusion, liquid phase diffusion, and surface diffusion along metal surfaces in a copper fleece-based anode. (b) GITT measurements of effective solid-phase diffusivity of foil (square) and fleece (circular) anodes of different thicknesses. The effective solid-phase diffusivity of a commercial LGM 50 cylindrical cell with an electrode thickness of approximately 85 m is included as (cross).

As a consequence of the studies reported here, Li^+^-ion diffusivity and electrical conductivity of electrodes could be enhanced by a novel design where the copper/active material interface is greatly enhanced. In the following, we report on replacing the traditionally applied copper foil of a graphite anode in a Li^+^-ion half-cell with an ultrafine copper fiber fleece inserted into the active material (Figure 3a) and comparing the Li^+^-ion diffusivity in foil- or fleece-based anodes. The copper fleece infiltrates the active material and is thus in direct, optimal contact with the active material and the electrolyte. Since the fleece is made of ultrafine copper fibers, it also provides more surface area per copper weight than a foil for the same weight. The Li^+^-ion diffusivity achieved in electrodes employing the copper fleece or the foil is quantitatively studied by GITT analysis of graphite anodes of different thicknesses and porosities (details are given in Supporting Information IV). Here, we focus on the graphite anode, where in today’s graphitic anodes, the active material layer is limited to less than 100 μm, mainly due to the sluggish lithium-ion diffusion. Du et al.^46^ and Suthar et al.^47^ reported that the capacity fade and high overpotential in thick anodes are mainly due to the ion diffusion limitation based on calculations and impedance measurement.

Two types of graphite anodes were prepared: (i) traditional anodes with a copper foil as the current collector, referred to as the foil anode (Figure 3a, left), and (ii) anodes with a copper fleece, referred to as the fleece anode (Figure 3a, right). The electrodes were fabricated with different thicknesses ranging from 29 to 457 μm for copper foil-based anodes and from 280 to 1706 μm for copper fleece-based anodes (details are shown in Tables S3 and S4). In all cases, the electrode composition was identical: 90 wt % synthetic graphite, 3 wt % carbon black, and 7 wt % of PVDF-HFP. The copper fleece density and diameter of the ultrathin metal fibers (as shown in Figure S12) were identical for all fleece anodes: 445 mg/cm^3^ density and approximately 160 μm mesh size (as shown in Figure S14). Thus, in all fleece anodes, the copper fleece volume fraction is set to 4.8∼5.5% (Table S2). As a comparison, in a commercial cell anode (LGM 50), the copper foil volume fraction is 11.8%. In comparison, the fleece electrodes use half of the copper amount than foil electrodes and therefore provide higher volumetric and gravimetric energy density (a detailed volumetric and gravimetric energy density comparison between foil and fleece electrodes is given in Table S5).

Figure 3b shows the GITT measurements on the graphite half-cells. In this study, we use the term “effective solid-phase diffusivity” to refer to this electrode-thickness-dependent solid-phase diffusion constant. The insufficient Li^+^-ion supply through the pores of the thick electrodes alone (liquid-phase diffusion) affects the solid-phase diffusion. For foil anodes, the effective solid-phase diffusivity drops linearly on a logarithmic scale with increasing electrode thickness (Figure S20). The slope of the linear drop increases with material density, i.e., reduced active material porosity. For electrode thicknesses beyond 120 μm, the effective solid-phase diffusivity drops below 10^–11^ cm^2^/s. In the case of fleece electrodes (circular dots), the logarithmically plotted effective solid phase diffusivity also decreases linearly but at a much more moderate rate also shown in Figure S20. At higher material densities, the plotted lines exhibit a parallel shift to lower effective solid-state diffusivity values. Table S4 summarizes the active material filling amounts in different fleeces used in this study. The thickest anode that we tested was 1.7 mm thick with an electrode density of 0.8 g/cm^3^. Also, with an electrode density of 1.2 g/cm^3^, the effective diffusivity does not drop below 10^–11^ cm^2^/s at a thickness of ca. 1.2 mm. Remarkably, the extrapolation of the fleece electrode data suggests that thinner fleece electrodes act like foil electrodes. This occurs when the thickness of the fleece electrode reaches half the diameter of the mesh size at about 80 μm. For comparison, the effective solid-phase diffusivity of an LGM 50 cylindrical cell with an electrode thickness of approximately 85 μm and an effective diffusivity of 1.48 × 10^–11^ cm^2^/s is included in Figure 3b.^28^

In thick electrodes, a long diffusion path and small electrode porosity hinder Li^+^-ion liquid-phase diffusion and impact the local availability of Li^+^-ions at active material particles, as described by Kremer et al. and Gao et al.^1,12^ This affects the solid-phase diffusivity within the graphite particles. The GITT data in Figure 3b show that the impact of the anode thickness on effective solid-phase diffusion in foil and fleece anodes differs greatly. In foil anodes, liquid-phase diffusion is the only pathway for Li^+^-ion distribution within the active material to reach all graphite particles. The fact that effective solid-phase diffusivity in fleece electrodes with the same material density and larger thickness is much greater suggests that fleece anodes must offer a second and highly effective path for assisting the distribution of Li^+^-ions throughout the active material and thus enhance local Li^+^-ion supply to graphite particles throughout the anode.

The ion flux, , through the active material under the influence of a spatial ion concentration gradient and an electric field, is described by the Nernst–Planck equation^48^:
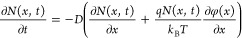
1with *D* being the diffusion constant, *N*(*x*,*t*) the ion concentration, *q* the charge, *k*_B_ the Boltzmann constant, *T* the absolute temperature, and φ(*x*) the electrical potential.

In foil batteries, the Li^+^-ions are transported through the liquid-electrolyte-filled pores of the active material to the solid graphite particles. The liquid-phase transport of Li^+^-ions then changes to solid-phase diffusion into the solid graphite particles (Figure 3a, left). Adopting the steady state condition ( = ϕ = const.), the spatial distribution of the Li^+^-ion concentration can be solved as
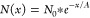
2with *N*_0_ being the Li^+^-ion concentration at the separator and *A* being the distance from the separator to the site in the electrode where *N* = *N*_0_/*e*.

The electrical potential is solved as
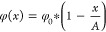
3

The potential is set to be 0 at site *A*, and φ_0_ is the potential of the electrode at the separator. Then the Nernst–Planck equation becomes
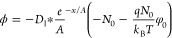
4

with D_l_ describing the Diffusion of Li^+^with D_l_ describing the Diffusion of Li^+^-ions via the liquid phase.-ions via the liquid phase. Therefore, the ion flux ϕ, which reaches the solid graphite particles, becomes a function of the electrode thickness, *x*. At distance *x* from the separator, the ion flux is reduced by . The reduction of the ion flux that reaches solid graphite particles causes a rate-limiting mechanism that governs the solid-phase diffusion within the solid graphite particles. Hence, the effective solid-state diffusivity *D*_s_^eff^ scales with the liquid phase diffusivity *D*_l_ and the electrode thickness *x* according to
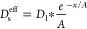
5
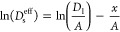
6

Therefore, the determination of the effective solid-phase diffusivity on the logarithmic scale as a function of electrode thickness *x* for foil anodes reveals a linear drop. Greater material density, i.e., reduced active material porosity, decreases intrinsic liquid-phase diffusivity *D*_l_. The distance at which the ion concentration drops to *N*_o_/*e*, i.e., *A*, is shorter for denser materials. Therefore, denser material causes a faster drop in the straight lines when scaling logarithmically against the anode thickness and confirms the experimental findings in Figures 3b and S20.

In the case of the fleece anode, the metal fleece infiltrates the active material. It provides a second path for ions to distribute into the active material, described by the diffusion constant *D*_m_ (the Li^+^-ion diffusivity along the metal fiber surface) (Figure 3a, right) in parallel to diffusion via the liquid phase, *D*_l_. In the case of thick electrodes, we approximate that the distribution of Li^+^-ions into the active material via the metal fleece is much more effective than via the liquid phase, i.e., *D*_m_ ≫ *D*_l_.

The graphite particles are located in the meshes of the metal fleece. Therefore, ions first diffuse via the metal fleece to the metal fleece meshes and then are transported to the graphite particles by liquid diffusion into the mesh. In this serially coupled ion flux prior to entry into the solid graphite particles, the diffusion constant in the Nernst–Planck eq 1 can be approximated to
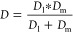
7

Thus, the ion flux through the liquid phase within the mesh size (prior to reaching the solid graphite particles) does depend on the porosity of the active material, i.e., active material density. In analogy to eq 4, the effective solid-state diffusivity *D*_s,f_^eff^ for metal fleece electrodes scales as
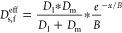
8with *B* being the distance from the separator in the electrode, where *N* = *N*_0_/*e* and the potential drops to zero. With *D*_m_ ≫ *D*_l_, the *D*_s,f_^eff^ can be approximated to
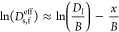
9

Since the fleece is assumed to add high Li^+^-ion mobility channels, the following accounts *B* ≫ *A*, i.e., Li^+^-ions penetrate much deeper into the electrode in the case of a fleece battery in comparison to a foil battery. Comparing eqs 6 and 9, the ion flux that reaches solid graphite particles on the logarithmic scale drops linearly with electrode thickness *x* in both cases. Since *B* ≫ *A*, the slope of the linear drop is much less steep for fleece electrodes than for foil electrodes (Figure 3b). Since the ion flux through thick fleece electrodes is mainly governed by diffusion along the metal fleece, and metal fleece parameters such as mesh size are not changed for the measurements, the slopes of the straight lines for the three different graphite densities are approximately identical (Figure 3b). Densifying graphite decreases *D*_l_ while metal fleece parameters *D*_m_ and *B* remain constant. Therefore, the parallel drop of the straight lines, as presented in Figures 3b and S20 for fleece anodes, is due to an increase in graphite density, whereas the steepness of the slope of the straight lines is determined by the copper fleece properties. In other words, the infiltration of the metal fleece homogenizes the ion concentration gradient along the electrode’s thickness significantly. Therefore, in fleece electrodes, the diffusion resistance is, in principal, independent of thickness. However, the ion diffusion from the metal fleece to the active material particles intercalation sites of Li^+^-ions in Graphite depends on the porosity of the active material and is seen by the parallel drop of the straight lines (Figures 3b and S20).

**Figure 4:**
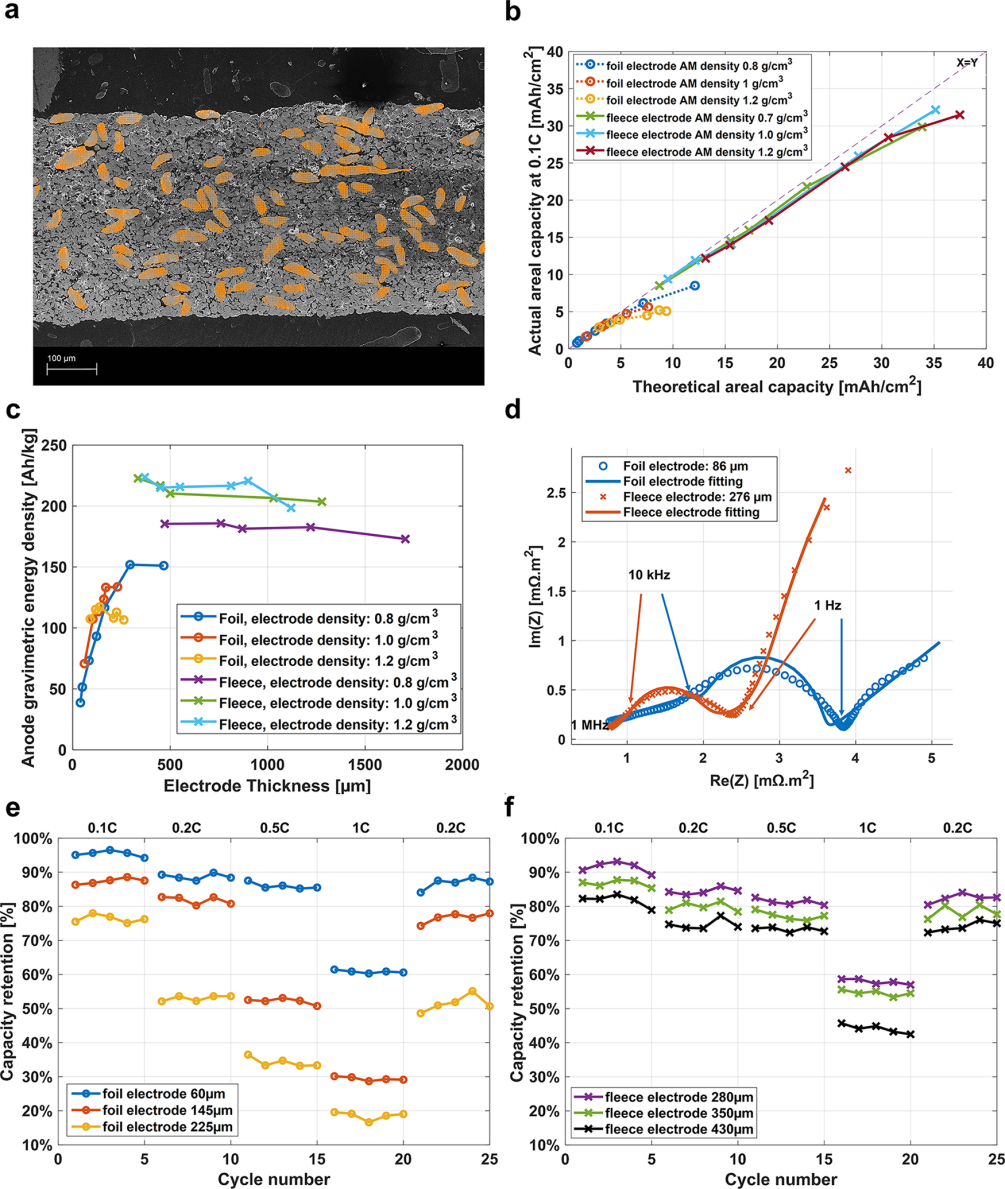
(a) SEM image of fleece electrode cross section (copper labeled orange); (b) theoretical areal capacity vs measured areal capacity at 0.1C; (c) gravimetric energy density of fleece and foil anodes (current collector mass included); (d) EIS data and the data fitting of foil and fleece electrodes; rate capability of (e) foil and (f) fleece electrodes of different thicknesses.

Eventually, the electrochemical properties of foil and fleece anodes of different thicknesses were tested in half-cell configurations. Figure 4a presents a typical cross-sectional SEM image of the fleece electrode with ca. 350 μm thickness. Figure 4b presents the comparison between the electrode’s areal capacity at 0.1C and their theoretical areal capacity (data listed in Tables S3 and S4) as measured by charge–discharge cycles. For foil electrodes, the experimentally accessible capacity at 0.1C deviates from the theoretical capacity with increasing thickness and graphite density. Increasing the electrode thickness or decreasing the porosity significantly affects the lithium-ion path and tortuosity of the foil electrode, making it impossible to access the theoretical capacity above a certain electrode thickness. On the contrary, the fleece electrodes are fully lithiated/delithated at 0.1C, regardless of thickness or porosity. Table S5 presents the areal capacity (at 0.1C), volumetric energy density, and gravimetric energy density of foil and fleece electrodes with different thicknesses and active material porosities. With comparable electrode porosity, the fleece electrodes provide higher volumetric and gravimetric density than foil electrodes. Figure 4c presents the foil and fleece anode gravimetric energy density (@ 0.1C). Due to the lower mass fraction of the current collector and efficient utilization of active material, the fleece anodes are able to provide up to 85% higher gravimetric energy density for 1.2 g/cm^3^ electrode density than foil electrodes. The energy density of foil electrodes increases linearly with the thickness of the electrode as the absolute contribution of copper to the weight of the electrode remains constant, but the added mass of active material increases. However, at an electrode thickness greater than about 180 μm, the measured energy density saturates because the additional active material added within thick layers cannot be fully reached by Li^+^-ions. In contrast, for fleece electrodes, the energy density is constant with an increasing electrode thickness because the volume ratio of copper to active material is constant. Therefore, the mass of the active material and the mass of copper increase by the same ratio. However, the copper fleece allows Li^+^-ions to be delivered to the entire electrode and allows sufficient charging of even very thick electrode layers.

A copper fleece improves the DC electrical conductivity of the electrodes (Table S6). It also reduces the internal resistance as measured by impedance measurements (EIS) and presented in Figure 4d of the foil (86 μm) and fleece electrode (276 μm) in a half-cell format. The EIS data fitting is based on an equivalent circuit (Figure S21) with a constant-phase element (CPE) instead of a finite-length Warburg (FLW) element because the data do not extend to low enough frequencies to demonstrate the finite (low-frequency) character of the Warburg element. As shown in Figure 4d, specific internal resistance as measured at 10 kHz is only 1 mOhm m^–2^ for fleece electrodes versus 1.8 mOhm m^–2^ for foil electrodes. The up to 55% reduction of specific internal resistance for fleece versus foil electrodes is expected partly due to the approximately three times larger copper surface area, which reduces the resistance between the copper surface and active material particles, although the thickness of the fleece electrode is 3 times larger. The foil electrode has higher impedance than the fleece electrode as measured between 10 kHz and 1 Hz, indicating that the charge-transfer resistance of the fleece electrode is smaller compared to the foil electrode. We further argue that the enhanced Li^+^-ion diffusion by the metal fleece optimizes the ion distribution in the electrodes. Based on the Butler–Volmer equation (eq 10), the overpotential (η_overpotential_) induced by the charge-transfer resistance depends on the lithium concentration within the AM particle (*c*_s_) and the surrounding electrolyte (*c*_e_). A homogeneous *c*_e_ distribution decreases the overpotential induced by charge-transfer resistance.
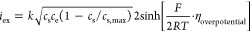
10Here, *F* is the Faraday constant, *R* the gas constant, *T* the temperature, *c*_s,max_ the maximum lithium concentration of AM particles, *k* the Butler–Volmer reaction rate, and *i*_ex_ the exchange current density.

EIS results also show a significant difference in the diffusion process between foil and fleece electrodes. At low frequency range (0.1 to 1 Hz), there is a significant difference of the ‘tail’ slope of foil and fleece electrodes, indicating that metal fleece electrodes enhance the ion diffusivity compared to foil electrodes, although the fleece electrode is 3 times thicker than the foil electrode.

Due to the large variation of electrical and diffusive resistance between foil and fleece electrodes, the half-cells perform quite differently at high charging rates, i.e., C-rates. Figure 4e,f compares the rate performance of fleece and foil electrodes in half-cell format (graphitic anode against metallic lithium); the charging protocol is CC–CV charging and CC discharging. The capacity retention is calculated based on the CC discharging capacity. Here, the thin foil electrode (60 μm thickness) presents a good capacity retention even at high cycling rates, as expected for such thin electrodes. However, a drastic drop in reversible capacity is observed when the thickness of the foil electrode is increased to 145 and 225 μm (Figure 4e). For fleece electrodes, no strong thickness dependence on the rate capability is measured (Figure 4f). The capacity retention at high C-rates is very similar to the foil electrode with 60 μm thickness. Fleece electrodes of such great thicknesses as those shown here have an area capacity of ca. 10 mAh/cm^2^. Thus, 1 C current density is larger than 10 mA/cm^2^ which is beyond the expected critical current density for half cells.^49,50^ In this case, the charge transfer resistance of metallic lithium is large and the limiting factor in rate capability testing. In order to understand which factor (electrical resistance or diffusive resistance) is limiting thick anode performance, simulations based on the DFN model are carried out (details described in Supporting Information IV). These calculations (Figure S15) demonstrate that an increase in the anode electrical conductivity does not improve the electrochemical performance of thick anodes. Instead, the high diffusive resistance is the limiting factor. Therefore, the improved rate capability in fleece electrodes is mainly because of the diffusion rate enhancement within the electrodes caused by the copper fleece.

## Conclusions

We quantitatively described a microfluidic experiment that demonstrates enhanced Li^+^-ion mobility along a copper surface immersed in a liquid electrolyte when increasing the electrical potential on the metallic surface. The such induced electrical double layer—also called the Helmholtz layer—attracts Li^+^-ions to the copper surface and reduces the ion solvation shell and the ion diffusion resistance. Molecular initio-dynamic simulations of the process reveal up to 56 times higher ion diffusivity along the charged copper surface than in the electrolyte.

Including such a charged copper surfaces in an anode electrode, i.e., the formation of a 3-dimensional copper fiber-graphite composite, is beneficial for the electrochemical performance of an anode. Such a composite enabled electrochemically functional anodes with a thickness of more than 1.2 mm and an area capacity of approximately 32 mAh/cm^2^ as measured by GITT in half-cells. In summary, the ultrafine copper fleece adds an additional channel to the electrode for enhancing electrical conductivity and Li^+^-ion diffusivity, thereby enabling functional ultrathick anodes of unreported thickness. The ultrafine metal fleece that replaces metal foil as the current collector in electrodes takes up less space and weighs less, i.e., the weight ratio of current collector and active material is decreased from 42 to 23%. In fact, the weight of the metal fleece is a factor of 2 smaller than the weight of a copper foil per given capacity; thus, the energy density per weight of the cell is greatly enhanced through replacing the traditional copper foil by a copper fleece.

## Method

### Microfluidic Device Assembly

In brief, a copper layer was deposited onto the silicon wafer by E-beam evaporation. Microfluidic channels were constructed by micropatterning a layer of negative photoresist (SU-8 3000 series) by using direct writing laser lithography. The resulting channels were sealed at the top through chemical bonding of a thin PDMS sheet activated by nitrogen plasma treatment. PDMS-based tubing connectors were bonded to the inlets and outlets. Finally, a copper wire was soldered onto the wafer’s copper surface to allow for the application of an electrical potential. A detailed protocol describing device assembly and a troubleshooting guide are available in Supporting Information I.

### Electrolyte Description

Ultrapure LiPF6 (99.8%, Sigma-Aldrich, Germany) and the electrolyte solvent (ethylene carbonate (EC): dimethyl carbonate (DMC) 50:50, Sigma-Aldrich, Germany) were mixed in an argon atmosphere glovebox with low H_2_O and O_2_ content (<0.1 ppm). LiPF_6_ was baked at 80 °C for 48 h under a vacuum. The glassware was cleaned with H_2_O_2_:H_2_SO_4_ at a ratio of 1:3, washed with deionized water in an ultrasonic bath, and dried under a vacuum for 24 h.

### Raman Spectrum Acquisition, Concentration Calibration, and Noise Reduction

A scanning near-field confocal Raman microscope (AlphaSNOM, Witec, Germany) equipped with a 532 nm laser (max. power: 40 W) and a glass objective with 50× magnification corrected for aberrations (Zeiss 50×/0.55 DIC LD, Zeiss, Germany) provided an ∼1.2 μm spot size at 9 mm working distance. Small calibration chambers were designed and built from photoresist onto a copper-covered Si-wafer (SU-8 3025). Also, a thin sheet of PDMS was bonded to seal the channel. Electrolytes with a Li^+^-ion concentration of 0–2 M were injected, and spectra were recorded at 50% laser power. Each separate measurement consisted of an average of 10 spectra, each collected using a 0.5 s exposure time. Measurements were repeated 5 times. The MATLAB script was used for background removal. Fluorescence background noise was too large to be negligible because the lithium salt in the solvent evokes greater fluorescence. First, measurements were calibrated by normalizing the Rayleigh peak intensity to account for slight differences in the laser intensity between measurements. In a second step, the data were normalized by comparing the spectral intensities between the Raman bands at 720 and 733 cm^–1^. These represent the O–C–O bonding in the presence of or without the presence of Li^+^. Lithium concentrations were visualized by Raman spectroscopy. Details on data cleaning and the calibration process, as well as results, are given in Supporting Information II.

### Microfluidic Device Operation and Operando Raman Line Scan Measurement

To conduct diffusion measurements between a lithium-ion-rich phase and a lithium-ion-poor phase, a 1 mol/L LiPF_6_ solution in 50:50 EC/DMC as well as a 50:50 EC/DMC solution without LiPF_6_ were prepared. The solutions were loaded into gastight glass syringes (SKU:HM-1001 Darwin microfluidics, Germany), which were connected to a needle (0.4 × 19 mm, BD Microlance 3, Spain) and polytetrafluoroethylene (PTFE) tubing (OD × 0.32 mm, Darwin microfluidics, Germany). After the tubing was purged, the syringes were connected to the microfluidic device via the PDMS inlet holes. The laminar flow was controlled by a syringe pump (Pump II Pico Plus Elite, Harvard Apparatus, USA), and the flow rates of both solutions were set at the same value. The flow rate was selected based on the Reynolds number and diffusion rate (Supporting Information II). After the entire channel was wetted, the leakage indicator channels were checked to exclude any leakage at the PDMS-bonded seals. For experiments where an electrical potential was applied to the copper surface by a source meter (Keithley 2615 A Source meter), grounding electrodes were connected to the needles of the syringes, whereas the active electrode was connected to the copper wire on the copper substrate. The source meter applied a constant voltage during the entire measurement. To avoid any electrochemical reactions, the voltage range we chose was between −500 and 500 mV.

The whole device was fixed on a piezoelectric stage to ensure that the microfluidic channels were in the horizontal plane. A test line scan along the *z*-axis was carried out to locate the PDMS and copper surface. Next, the focus point was fixed to the central line of the channel. Afterward, a 1-D line scan along the *y*-axis was carried out with the same settings for calibration purposes. Due to the presence of laminar flow, the impact of the laser power on electrolyte degradation and temperature was negligible, thus allowing the adoption of a higher output power. Raman spectra were obtained in a line scan along the channel width at 40 different points with a 5 μm distance between the points. The number of independent Raman measurements at each point was, on average, 10 per sample. A confocal Raman microscope uses a pinhole aperture to get rid of out-of-focus signals, thereby enabling the collection of volume information with a defined depth. The microfluidic device design also featured a ruler next to the leakage indicator channel (Supporting Information II) in order to locate the line scan starting and end points and thus ensure repeat accuracy. After calibrating the device as described above, the concentration was “visualized”, and the apparent Li^+^ diffusivity was calculated based on Fick’s law.

### PFG-NMR

In order to verify the reliability of the Raman and microfluidic measurements, pulsed field gradient NMR (PFG-NMR) measurements of Li^+^ diffusion coefficients were performed with a Bruker AVANCE III 400 MHz spectrometer and a Bruker wide-bore magnet. The shape of the gradient pulses was given by a half-sine function, and the diffusion time between the two gradient pulses of a pulse sequence was fixed at 10 ms. The amplitude of gradient pulses G was varied in 16 steps up to 10.55 Tm-1 for the bulk electrolyte.

### MD Simulation

MD simulation is implemented in the Material Studio (MS). The COMPASS III force field was used to optimize the atoms and charges. Simulation details are in Supporting Information III.

### Fleece Fabrication, Electrode Preparing, Cell Assembly

Fine metal fibers were produced with a melt spinning technique to produce fibers with a width of approximately 40 μm and a thickness of about 15 μm. The fine metal fibers are then cut to ∼7 mm in length, and the fleece is fabricated by dispersing the cut fibers in paraffin oil and then sintered them to a mechanically stable fleece under inert gas (argon) at 650 °C for several seconds. The areal loading of fibers and the thickness of the fleece were calculated geometrically to achieve a certain mean pore diameter within the fleece.

The sintered fleeces were filled with a graphite-based slurry containing 90 wt % synthetic graphite (Targray, product number: SPGPT802), 3 wt % carbon black (Alfa Aesar, 90+% trace metals basis, CAS: 1333-86-4), and 7 wt % PVDF-HFP (Sigma-Aldrich, CAS: 9011-17-0). All components were dissolved or dispersed in acetone (Alfa Aesar, 1 wt % EMK) with a solid to liquid ratio of 1:2. The copper fleece was dipped into the slurry for 60 s, during which it was treated with ultrasound to get the air bubbles out and achieve a perfect filling of the fleece with active material slurry. The coated fleece was dried in hot air (80 °C) and afterward calendared to the defined porosity with a speed of 5 m min^–1^ at a temperature of 160 °C. The electrode was vacuum-heated at 100 °C to get rid of H_2_O and other residuals before being transferred to a glovebox.

Round electrodes were punched out of the original electrode material (14 mm in diameter) and transferred to a glovebox. The electrodes were soaked in the electrolyte for 30 min (1 M LiPF6 in EC: DMC 50:50 (volumetric); Sigma-Aldrich, Germany). For half of the cells, a metallic lithium coin acted as an anode and lithium source, separated by a glass fiber separator (Whatman 934-AH). All cells were assembled into state-of-the-art coin cell setups (MTI Corp.) with wave springs and separators fitted to the sizes of the electrodes. Afterward, the cells were sealed with an electrical coin cell crimper, stored for 24 h at room temperature to achieve electrolyte wetting, and then prepared for testing.

The conventional graphite anode was built with the same slurry recipe, coated on a 12 μm copper foil using the doctor blade method, and then calibrated to the desired porosity and thickness. The preparation and assembly processes were the same as those for the fleece electrodes. Detailed information and characterization are listed in Supporting Information IV.

### Micro CT Reconstruction

3 mm diameter electrode discs were punched out from the cathode material sheet and mounted on a special pinhead sample holder. Next, they were scanned using a lab-based μ-CT system (Skyscan 1272, Bruker, Germany). An X-ray source with 70 kV source voltage and a 85 μA source current was used with 586 ms exposure time. A 360° rotation scan using 0.2° rotation steps was carried out for the three-dimensional reconstruction of the electrode. A voxel size of 1 μm 3D volume of the electrode was reconstructed and then imported into a voxel-based analyzing commercial software package (Geodict, Math2Markt, Germany). Multiphase segmentation was carried out based on the Otsu algorithm, and then the interfacial area between pores and active material was calculated and used for GITT measurements.

### Electrochemical Measurement

Half-cell testing was performed in a coin cell using an SP-150 Potentiostat (Bio-Logic). After assembly, the cell underwent two SEI formation cycles at C/10 CC–CV charge in a voltage window from 12 mV–1.5 V. OCV, theoretical capacity and solid phase diffusivity were determined using the GITT. Toward this end, the half-cell was charged at C/20 for 12 min, followed by a 2h relaxation period. This pulse-relaxation cycle was repeated 100 times until the cell was fully charged. A detailed description of the effective diffusivity calculation is given in Supporting Information IV.

Potentiostatic EIS was carried out to determine the foil resistance and electrode electrical conductivity at different SOC. The applied frequency range is 0.1 Hz to 1 MHz with an amplitude of 5 mV. The data fitting with an equivalent circuit model is carried out in MATLAB.

## Supporting Information Available

Microfluidic device fabrication protocol; Raman micro spectroscopy calibration, operating, and diffusivity calculation; MD simulation; fleece and electrode characterization; and GITT and μ-CT reconstruction (PDF)
