# Supplementary material for: Enhanced Ion Mobility in Helmholtz Layer Enabling Ultrathick Electrodes
Source: ACS Nano. 2025 Apr 28;19(18):17917–28. doi: 10.1021/acsnano.5c04343 (PMC12080375; doi:10.1021/acsnano.5c04343)
Supplement: Supplementary file 1 — nn5c04343_si_001.pdf [file nn5c04343_si_001.pdf]

Enhanced ion mobility in Helmholtz-layer enabling ultra-thick electrodes

Yuanzhen Wang[†,‡], *Florian Aubermann*[†,‡,₴], *Joachim P. Spatz*[†,‡,₴,*]

[†]Max Planck Institute for Medical Research @ Bildungscampus Heilbronn, Dept. of Cellular Biophysics, D-74076 Heilbronn, Germany

[‡]Heidelberg University, Institute for Molecular Systems Engineering and Advanced Materials, D-69120 Heidelberg, Germany

[₴]Max Planck School Matter to Life @ Bildungscampus Heilbronn, D-74076 Heilbronn, Germany

* Correspondence: spatz@mr.mpg.de

Supporting Information I: Microfluidic device fabrication protocol

Step 1: Copper deposition on 4" silicon wafers by E-beam evaporation

Copper deposition on 4-inch silicon wafers was performed using an E-beam evaporation process (E-beam evaporator Pfeiffer Vacuum Classic 500). The wafer was assembled in the evaporation chamber, argon plasma applied to clean the surface, and the whole chamber was evacuated to 1.18 e-7 mbar. Afterwards, 5 nm chromium and subsequently 100 nm copper were deposited on the Si-wafer with a rate of 2 Å/s.

Step 2: Spin Coating of the SU-8 3000 photoresists

Copper-coated Si-wafers were cleaned using acetone and isopropanol. The wafers were heated to 110°C and cleaned with nitrogen gas. The hot wafers were completely covered with SU8 photoresist and spin-coated (Laurell Technologies Corp., USA) to accomplish specific channel thicknesses. Each spin coating run consisted of a spread cycle at 500 rpm for 10 s (100 rpm/s acceleration) and spin cycles at various speeds for 30 s (excluding acceleration time at 300 rpm/s) according to **Table S1**. Coated wafers were soft-baked for 3 min at 65°C and 5 min at 95°C. This procedure maximizes photoresist adhesion to the copper surface during development.

Height [µm]	SU8-3000 photoresists	Speed [rpm]
10	SU8-3010	3000
20	SU8-3025	4000
30	SU8-3025	2700

Table S1: Parameters of the spin coating protocol.

Step 3: Micropatterning by laser lithography

Microfluidic devices were designed using the QCAD-pro application (RibbonSoft, Switzerland) and transferred into the photoresist layer on the coated wafers by laser lithography using the µPG

101 Micro Pattern Generator (Heidelberg Instruments, Germany) in writing mode IV at a maximum power of 70 mW with 100%-pixel pulse duration and 1x2 energy mode settings. The post-exposure bake was conducted for 1 min at 65°C and 5 min at 95°C. The wafer was allowed to cool down to room temperature and unpolymerized photoresist removed using the mr-DEV 600 developer (micro resist technology, Germany) for 12 minutes, during which the developer solution was exchanged once. Remaining traces of developer solution on the wafer were removed with isopropanol.

Step 4: Preparation of PDMS sheets

A thin PDMS (Sylgard 184, Dow Corning, USA) sheet was prepared by mixing the oligomer and curing agent at a 10:1 (w/w) ratio, followed by degassing. To ensure uniform sheet reproduction with regards to thickness, a 3D-printed casting frame with a cross-sectional area of 50 cm^2 was taped to a petri dish and filled with 1 ml of the PDMS mixture and subsequently cured at 70°C for at least 2 hours. This resulted in a sheet thickness of 1-2 mm. The PDMS sheet was removed from the petri dish, cleaned using 70% ethanol spray, dried with pressurized air, and protected from both sides with tape to avoid any dust contamination. After the sheet was cooled to room temperature, inlets and outlets were punched into the sheet with a 0.75 mm biopsy puncher (Darwin Microfluidics, Germany).

Step 5: Sealing microfluidic channels by PDMS sheet bonding

To seal the microfluidic channels, the previously prepared PDMS sheet was chemically bonded to the SU-8 photoresist layer. To this end, the tape from the bottom side of the PDMS sheet was removed and the surface activated by exposure to low-pressure ultra-pure nitrogen plasma (200 W, 0.4 mbar, 60 s; PVA TePla 100, PVA TePla, Germany). The sheet was positioned on the photoresist layer, such that the inlet and outlet holes aligned, and bonded for 30 min at 100°C. To improve bonding, weights were applied on the PDMS sheet.

Step 6: Bonding of tubing connectors

To facilitate the connection to appropriate tubing, thicker PDMS blocks (4-5mm) were produced and equipped with inlets and outlets. The top PDMS surface of the PDMS-wafer composite as well as the PDMS tubing connector were exposed to low-pressure oxygen plasma (200 W, 0.5 mbar, 30s; PVA TePla 100, PVA TePla, Germany) and bonded together (2h, 80 °C).

Step 7: Soldering of copper wires to the wafer's copper surface

Copper wires were soldered to the copper surface of the microfluidic device with a low melting alloy (51IN-32.5BI-16.5SN, Indium Corporation of America, USA) to allow the application of an electrical potential.

Troubleshooting:

In the case of insufficient adhesion of the photoresist to the copper-coated wafer and/or the appearance of wrinkles (**Figure S1**) during development:

- Different soft baking durations should be tested. Depending on the hot plate used and the thickness of the resist, the optimal time can vary.
- Stronger exposure doses during photoresist patterning increases adhesion. We selected the highest possible dose on our device.

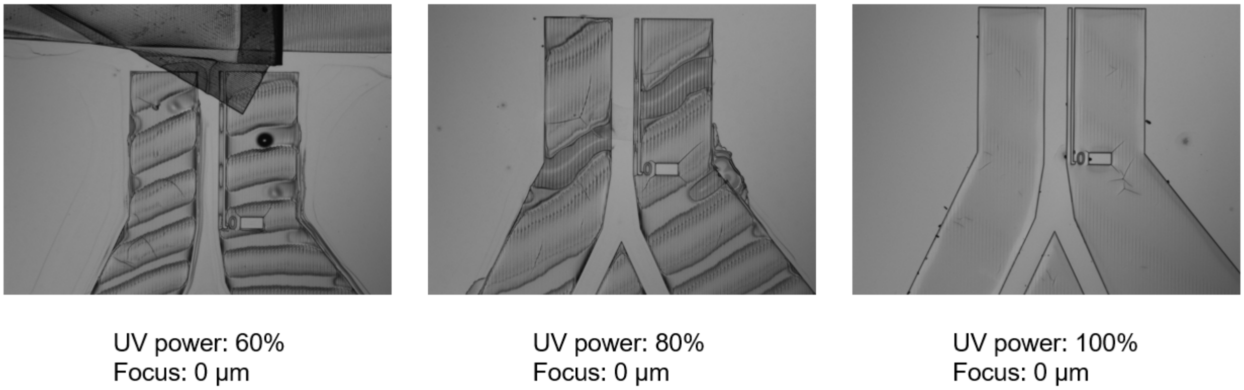

Figure S1: Adhesion is impacted by the exposure power and focus point.

The microfluidic device design also included two leakage indicator channels alongside the main channel (**Figure S2**), which fill with liquid droplets in the case of leakage. A ruler is placed next to the leakage indicator channel to facilitate reproducible measurements.

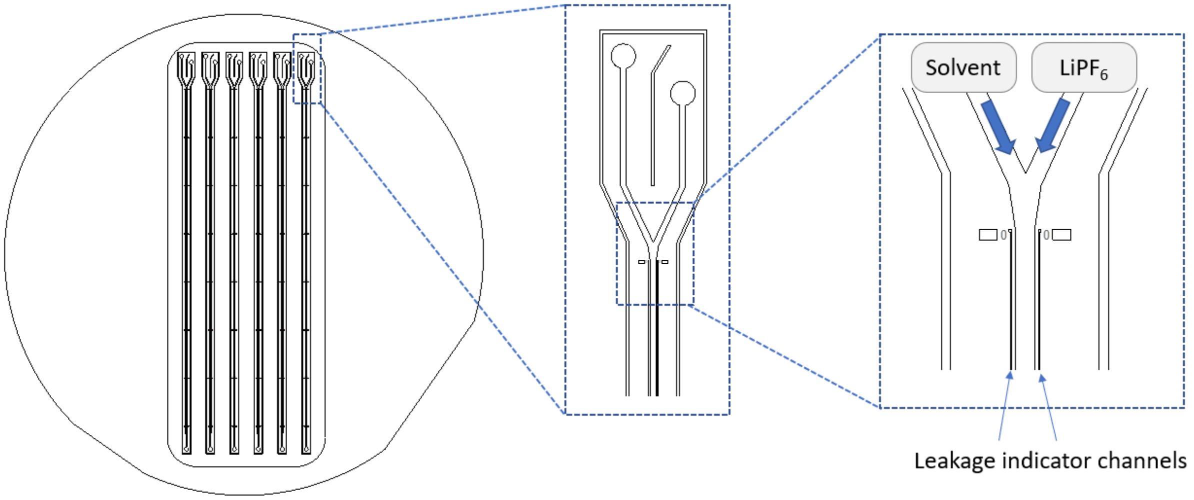

Figure S2: Main channel and leakage indicator channel design.

In case the copper surface changes color during baking steps:

- A change in color of the copper surface is an indication of oxidation, which is accelerated at higher temperatures. Baking under inert gas or at lower temperatures can prevent oxidation during baking.

If the thin PDMS sheet does not bond to the photoresist:

- Remember to avoid touching the photoresist or PDMS surface, as surface contamination and dust particles will decrease bonding performance.
- Make sure that no hard-bake was performed as it is usually recommended by the manufacturer of the photoresist. This will decrease the potential of the photoresist to react with the plasma activated PDMS.

- The nitrogen plasma applied to the PDMS sheet needs to be very pure. If there is any contamination of oxygen in the plasma, hydroxyl instead of amine groups will be preferentially formed on the surface and only amine groups will be able to bond to the photoresist layer.
- The pressure during incubation in the oven after bonding might not be high enough.

Supporting Information II: Raman micro spectroscopy calibration, operating and diffusivity calculation

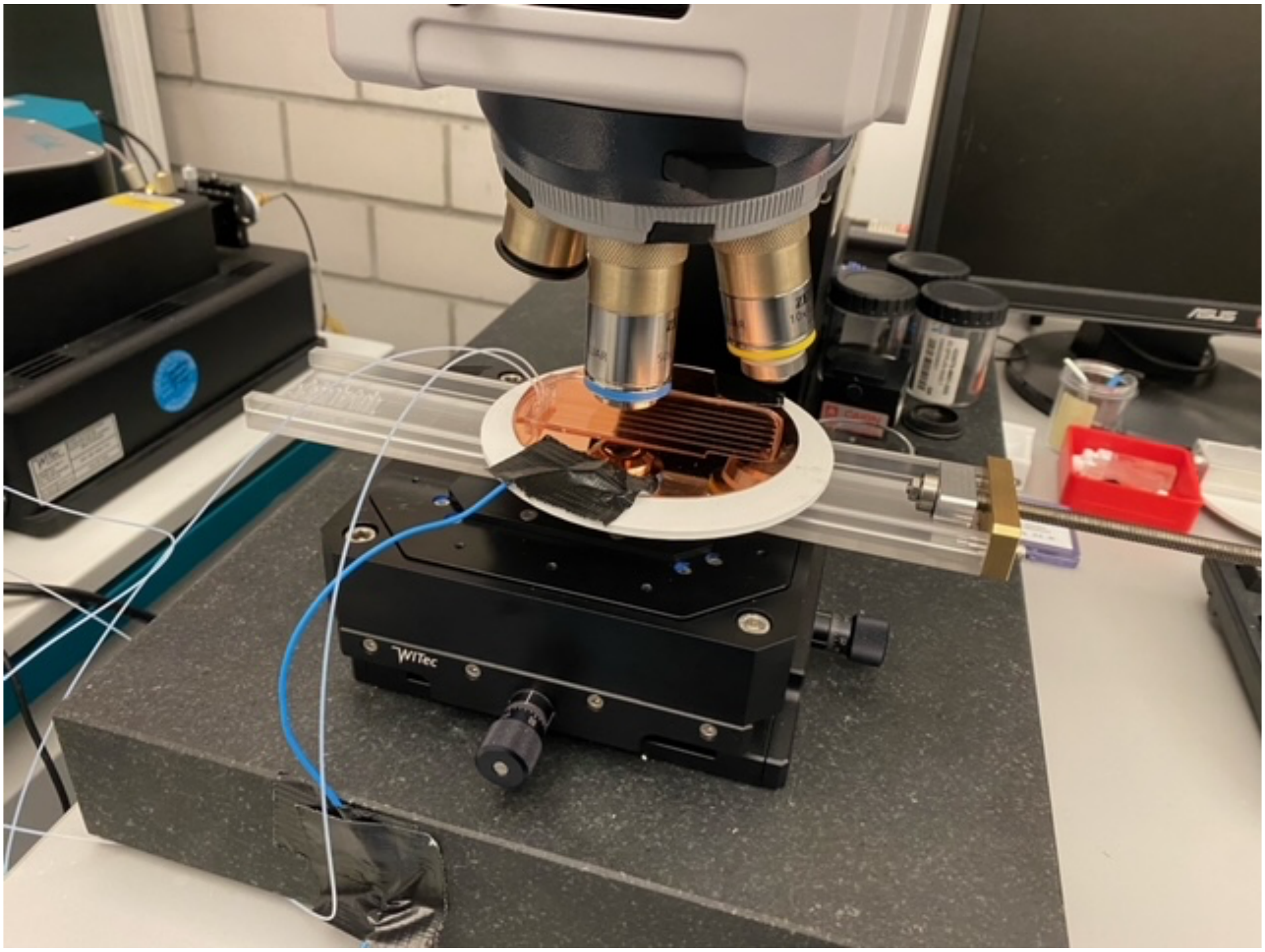

Figure S3: The experimental setup: The microfluidic channel was built on the copper-coated wafer. The organic solvent with or without lithium salt was connected to a grounding electrode and injected. An offset voltage was applied to the copper. The microfluidic device was scanned from the top through to the PDMS layer. A glass objective with 50x magnification and corrected for aberrations was utilized to gather the signal.

Raman spectra characterization

EC & DMC solvent mixed with different amounts of $LiPF_6$ were characterized with Raman spectroscopy (**Figure S3**). At greater lithium concentrations the EC's O-C-O bond vibrational frequency was shifted, due to the strong coordination between lithium-ions and EC molecules. A second significant indicator of lithium salt concentration was P-F stretching. Fluorescence background noise was too strong to be negligible, due to the lithium salt in the solvent which enhanced fluorescence (**Figure S4**). An algorithm was developed to determine the correlation between the Raman spectra and lithium salt concentration.

Step 1: Fluorescence noise reduction

Measurements were first calibrated by normalizing the Rayleigh peak intensity to account for slight differences in laser intensity between measurements and then fluorescence noise was eliminated from spectra by applying an algorithm that removes the low frequency peak of the signal (**Figure S5**).

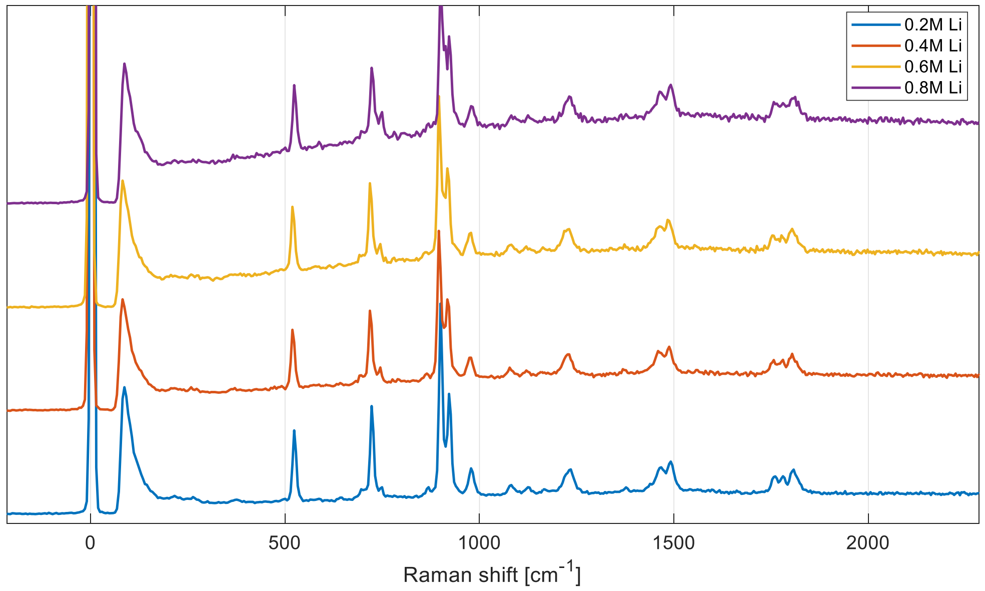

Figure S4: Raman spectra of electrolytes with different lithium-ion concentrations. The most significant Raman peaks were observed at 720 cm^{-1} (O-C-O) and 744 cm^{-1} (P-F).

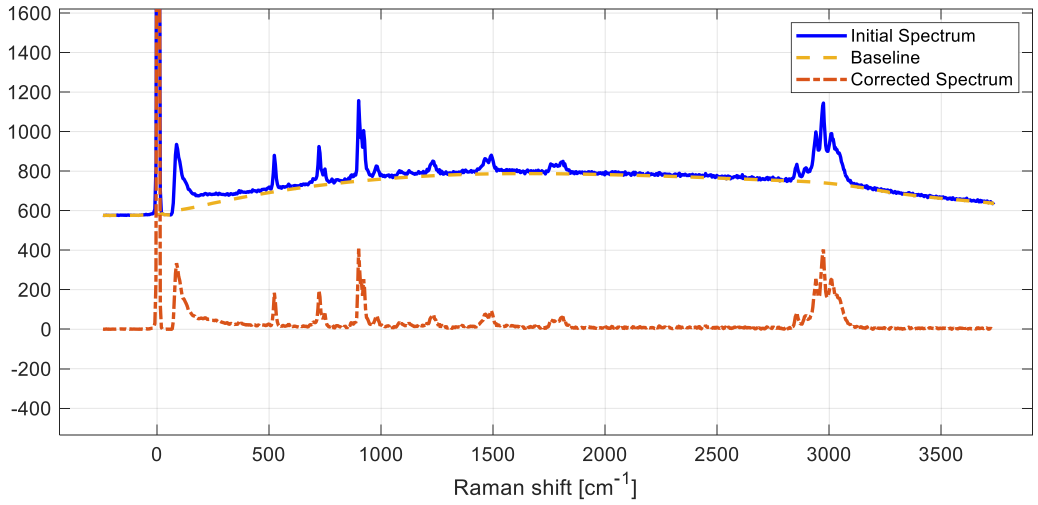

Figure S5: Fluorescence noise elimination.

Step 2: Visualizing lithium-ion concentration

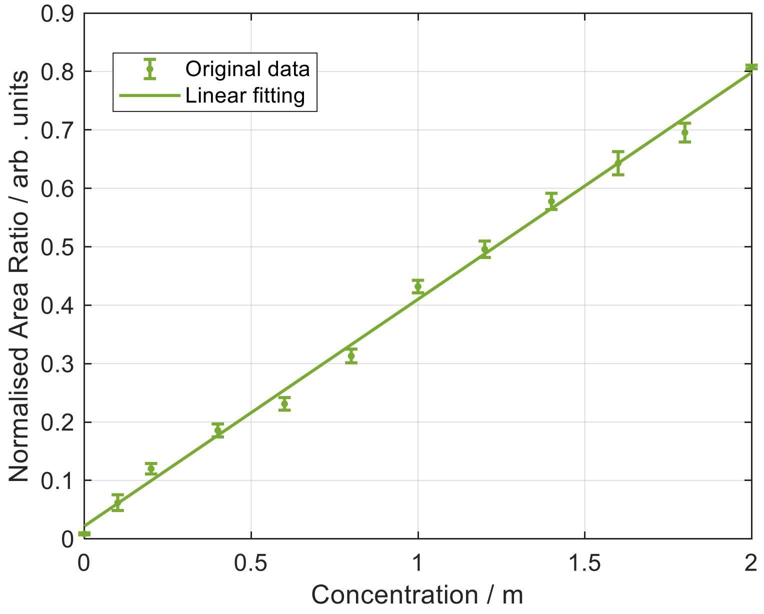

Figure S6: Normalized spectrum ratio at different Li salt concentration

As mentioned before, the most significant effects of lithium salt are O-C-O bond shifting and P-F bond stretching (**Figure 3b**). Here we observed that the integration of the two peaks is not linearly

correlated to the lithium-ion concentration, but the ratio between the two peaks displays a linear relationship with the lithium-ion concentration (**Figure S6**). The ratio R is calculated as

$$R = \frac{\int_{734\ cm^{-1}}^{754\ cm^{-1}} CCD - \int_{734\ cm^{-1}}^{754\ cm^{-1}} CCD_{noise}}{\int_{710\ cm^{-1}}^{730\ cm^{-1}} CCD - \int_{710\ cm^{-1}}^{730\ cm^{-1}} CCD_{noise}} \qquad \textbf{Equation S1}$$

CCD is the Raman shift intensity of the initial spectrum and CCD_{noise} is the fluorescence noise intensity. Using linear fitting (**Figure 1c**), the relationship between integration ratio and ion concentration is described as

$$c_{lithium} = 2.463 \times R - 0.00325 \qquad \textbf{Equation S2}$$

Flow rate determination

Laminar flow is the key prerequisite to characterize the diffusion coefficient measurement[1]. Thus, the selection of the flow rate is crucial for this measurement. On the one hand, the flow rate should be slow enough to guarantee a laminar flow condition and should allow enough time for the diffusion process to take place within the main channel. On the other hand, the flow rate should be fast enough that (i) convection flow is much greater than diffusive flow and (ii) a clear interface between different lithium-ion concentrations is generated at the Y-junction. The Reynolds number is calculated as

$$Re = \frac{\mu L}{v} \qquad \textbf{Equation S3}$$

Re is the Reynolds number, L is characteristic length, μ is flow speed and v is kinetic viscosity. Considering that the main channel width is much larger than the channel height, the characteristic dimension L is equal to the channel height.

Meanwhile, the flow rate should be large enough that diffusive flow along the x-direction is negligible. The Péclet number should be much larger than 1.

$$P_{eL} = \frac{L^2/D}{L/\mu} \qquad \textbf{Equation S4}$$

P_{eL} is the Péclet number and D is the diffusion coefficient. With this calculation we determined that the flow rate should be 1 µL/min, 2 µL/min and 3 µL/min for channels with a height of 10 µm, 20 µm and 30 µm, respectively. **Figure S7** shows the concentration gradient at 2 µL/min flow rate in a channel of 20 µm height. These parameters generate a clear concentration gradient at s0 while maintaining a low Reynolds number in the meantime, and establishes a clear interface at the Y-junction.

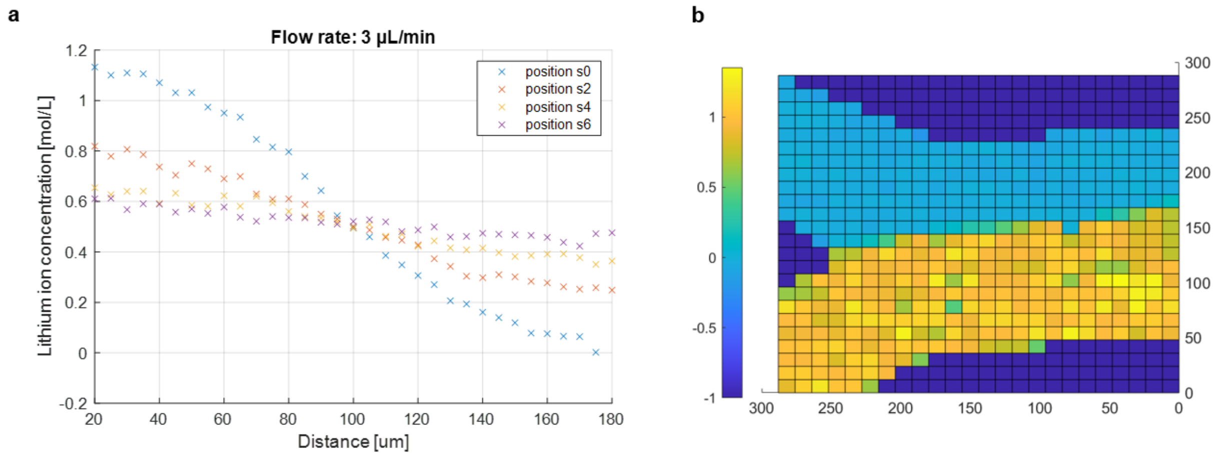

Figure S7: (a) The lithium-ion concentration gradient at 2 µL/min in a channel with a height of 20 µm. (b) 2D Raman scan of the Y-junction. The legend on the left indicates the lithium-ion concentration in mol/L (-1 indicates the PDMS wall).

Diffusion coefficient calculation

For quantifying the diffusion process, a convection-diffusion model[2] was applied to determine the apparent diffusion coefficient. The convection-diffusion model describes multicomponent diffusion between two co-flowing laminar flowing liquids in the microchannel.

When two channels join together, the two flows come into contact with each other and diffusion starts at the interface of the laminar co-flow. The convection laminar flow velocity profile in the rectangular duct along the y-axis can be described as

$$v(y) = \left(\frac{n+1}{n}\right)\left[1 - \left(\frac{y}{W/2}\right)^n\right]\bar{v} \qquad \textbf{Equation S5}$$

$\bar{v}$ is the mean velocity calculated by flow rate, W is the width of channel. The exponent n is dependent on the channel dimension

$$n = 1.7 + 0.5(\frac{H}{W})^{-1.4} \qquad \textbf{Equation S6}$$

H is the height of channel and the non-slip boundary condition is applied to the channel wall. Since the Péclet number is larger than 100, the diffusion along the x-axis is negligible. Diffusion along the y-axis is described by Fick's second law

$$\frac{\partial c}{\partial t} = D\frac{\partial^2 c}{\partial y^2} \qquad \textbf{Equation S7}$$

c is lithium-ion concentration, D is diffusivity and t is time. Here the diffusion time t can be substituted as

$$t = \frac{x}{v(y)} \qquad \textbf{Equation S8}$$

v(y) is the convection flow velocity along the channel calculated by Equation S5 and x is the flow distance along the x-axis. Therefore, Fick's second law can be re-written as

$$v(y)\frac{\partial c}{\partial x} = D\frac{\partial^2 c}{\partial y^2} \qquad \textbf{Equation S9}$$

In this convection-diffusion model, the second partial differential term $\frac{\partial^2 c}{\partial y^2}$ is the key part for the measurement. Also, a sufficiently small Δy is required. Therefore, the Raman line scan along the y-axis is implemented in 4 µm steps to accurately calculate the diffusion coefficient.

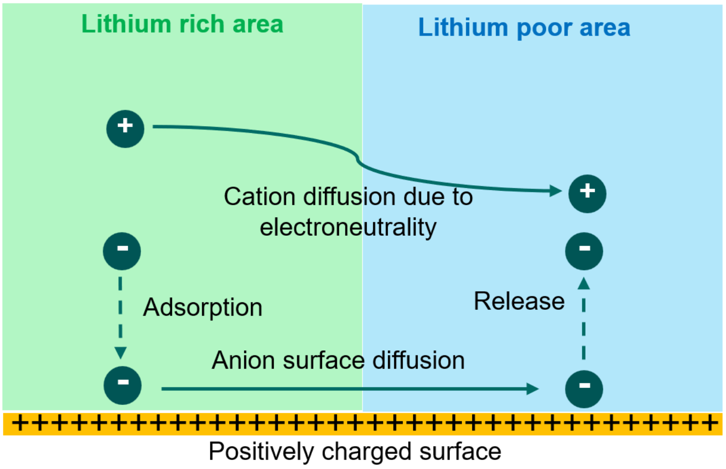

Figure S8: Schematic of the positively charged surface scenario, in this case the anions form the EDL and expand laterally along the surface, then release to the bulk in lithium poor area. However, in the bulk electrolyte, the electroneutrality must be kept thus the cations will be pulled to the lithium poor area and an enhanced lithium ion diffusion is observed in this case.

Supporting Information III: MD simulations

Molecular dynamic simulation

MD simulations were carried out using the COMPASS III force field. Simulations of bulk electrolytes were calculated at room temperature (293 K) with constant particle numbers and a constant volume. The electrolyte volume was 35.70 Å × 35.33 Å × 50.0 Å, with 30 Li^+, 30 PF_6^-, 225 EC molecules and 178 DMC molecules. This is equivalent to 1 M $LiPF_6$ in EC: DMC (50:50 v:v) electrolyte with a density of 1.195 g/cm^3. The electrode consists of 5 layers of copper atoms along the [1 1 1] direction and the surface area equals 35.70 Å×35.33 Å (see **Figure S9**).

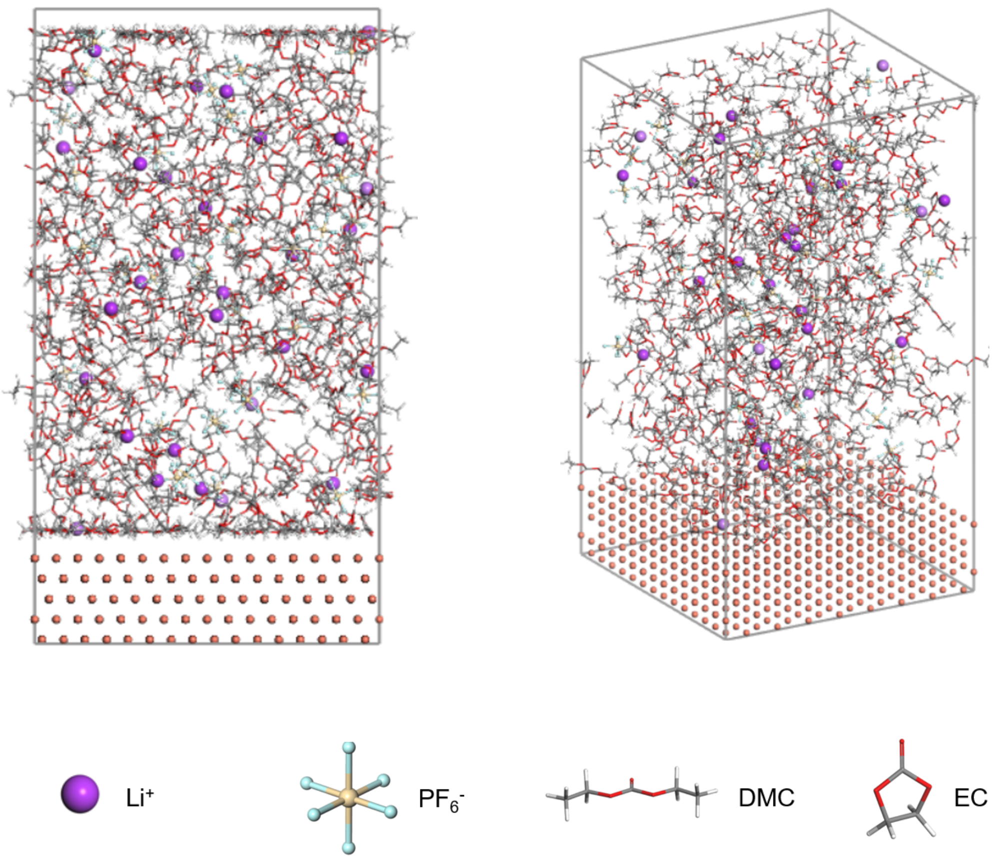

Figure S9: Solid electrolyte interface for the molecular dynamic simulation.

The COMPASS II force field together with atom-based Ewald electrostatic and van der Waals summation methods with convergence tolerance were selected for DFT calculations. The explicit solvent (DFT-CES) was applied. First, an equilibration run for 2 ns at room temperature with zero charge density condition on the electrode (1 fs time step) was performed, followed by a production run using the NVT ensemble for another 2 ns for a statistical analysis of the concentration distribution of each species. For the charged surface, 4 ns using the NVT ensemble at room temperature was performed to obtain the equilibrium state. When the species' concentrations were calculated at ~ 3 ns, the electric double layer (EDL) was converged. The surface and bulk concentrations are shown in **Figure S10**.

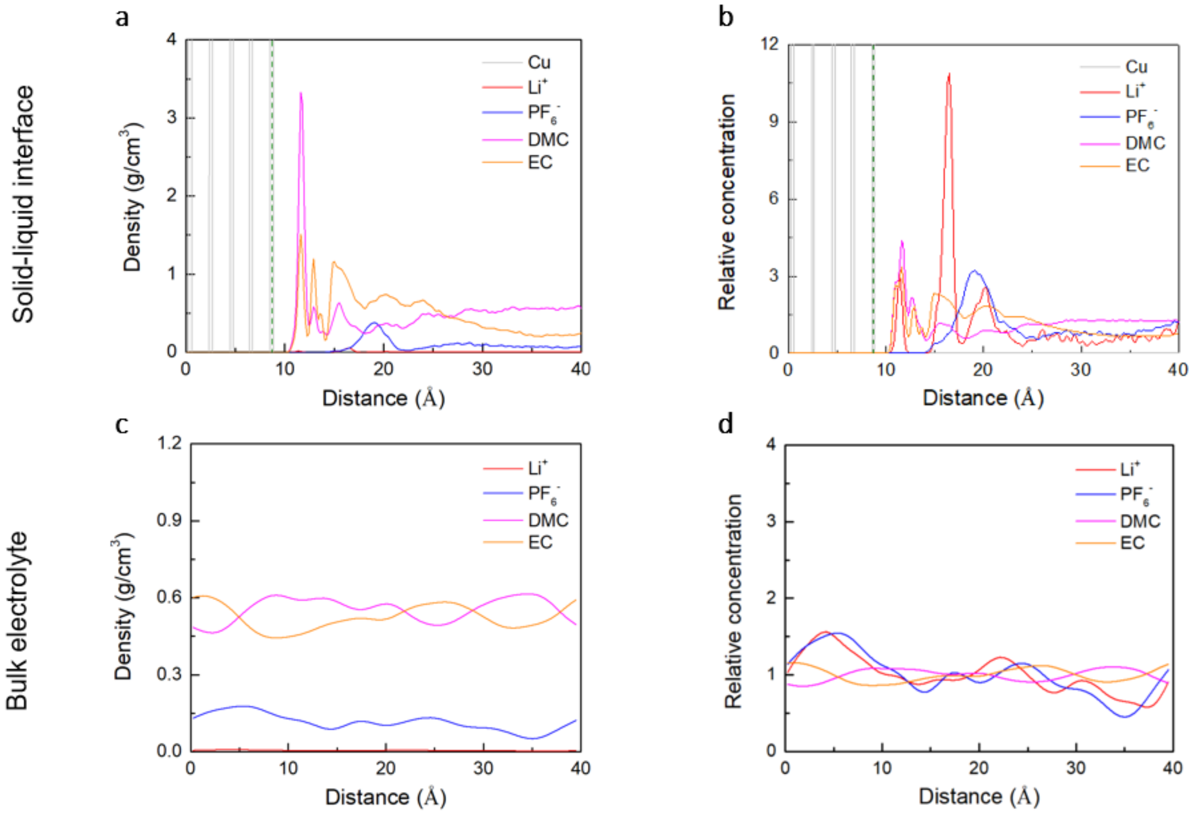

Figure S10: (a) Density distribution of molecules at the solid-electrolyte interface; (b) relative concentration of molecules at the solid-electrolyte interface; (c) density distribution of molecules in the bulk electrolyte; (b) relative concentration of molecules in the bulk electrolyte.

Figure S11 shows the number of coordinated Li^+-ion and bare Li^+-ion within EDL and bulk electrolyte based on molecule counting in a unit cell. Within the bulk electrolyte, most EC and DMC molecule are in 'free form', all lithium ions are coordinated with solvent molecule; within the EDL, when voltage applied the lithium ions start to accumulate and forms electrical double layer together with EC & DMC, at the meantime anions start to vanish, a portion of Li^+-ion are accumulated in 'coordinated form' but a large portion of Li^+-ion within EDL are in 'bare form', indicating that the SIPs, CIPs and ion-solvation aggregates are broken due to the electrostatic force.

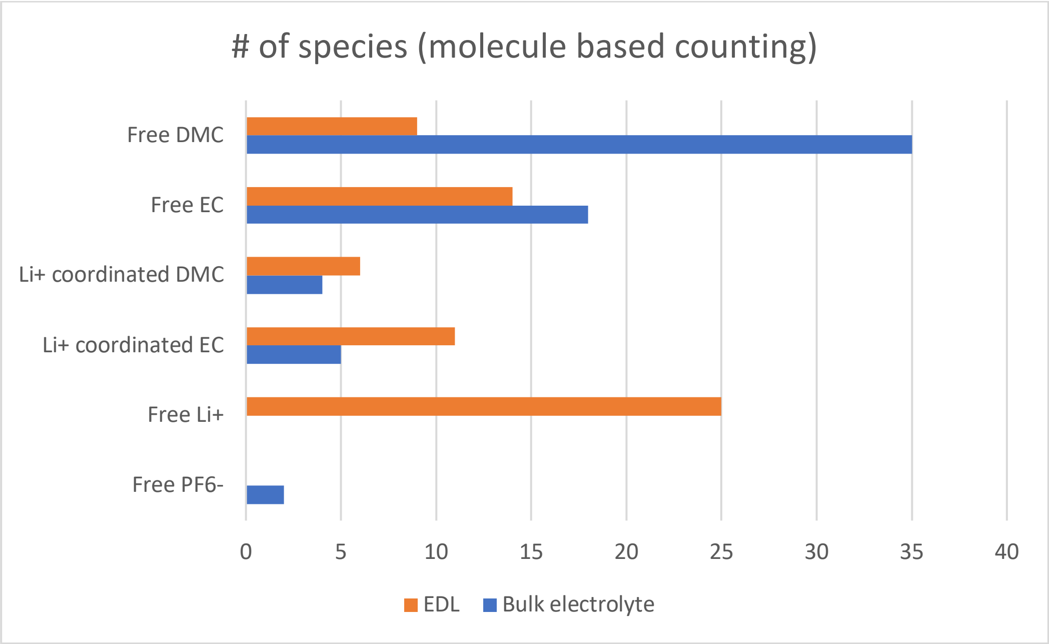

Figure S11: number of species (molecular based counting) in bulk electrolyte and EDL

As **Figure S10** and **Figure 4a** show there were two Li^+ concentration peaks near the copper surface within 10 Å above the charged surface. These ions were selected and tracked over time for the diffusivity calculations. The mean square displacement (MSD) of their trajectory was calculated for 700 ps. The diffusion coefficient was calculated using the Einstein relation

$$D = \frac{1}{6N} \lim_{t \to \infty} \frac{d}{dt} \left[\sum_i^N \left(r_i(t) - r_i(0) \right)^2 \right]$$ **Equation S10**

D is the diffusion coefficient, r_i is the center-of-mass position vector of particle i, N the total number of particles, and t is the time. Given a sufficiently long time, the mean square displacement starts to be proportional to the time according to the Fickian regime. The diffusion coefficient can be extracted by linear fitting.

Interfacial potential and charge density

The electrical double layer (EDL) is the result of the variation of electric potential near a surface. The relationship between the interfacial potential and the surface charge can be expressed by the Grahame equation, which is derived from the Gouy-Chapman theory[3] under the assumption of electroneutrality conditions:

$$\sigma = \sqrt{8 c_0 \varepsilon \varepsilon_0 k_B N_a T} \sinh\left(\frac{ze\varphi_0}{2k_B T} \right)$$ **Equation S11**

σ is the surface charge density, c_0 is the bulk electrolyte density, ε and ε_0 are the relative dielectric and vacuum dielectric constant, k_B is the Boltzmann constant, and φ_0 is the interfacial potential. During MD simulation, 50 mV was set as the interfacial potential. Thus 0.8 e/nm^2 was assigned as the charge density on the copper surface.

Supporting Information IV: Fleece and electrode characterization

Fiber and fleece characterization

The ultrafine copper fiber is fabricated by melt-spinning process. **Figure S12a** shows the optical image of the fibers and **Figure S12b** shows the SEM image of the fibers. As shown in **Figure S12b**, the fabricated fiber is in ribbon-shape, the average width of the fiber is ~38 μm and the average thickness of fiber is ~12 μm.

a b

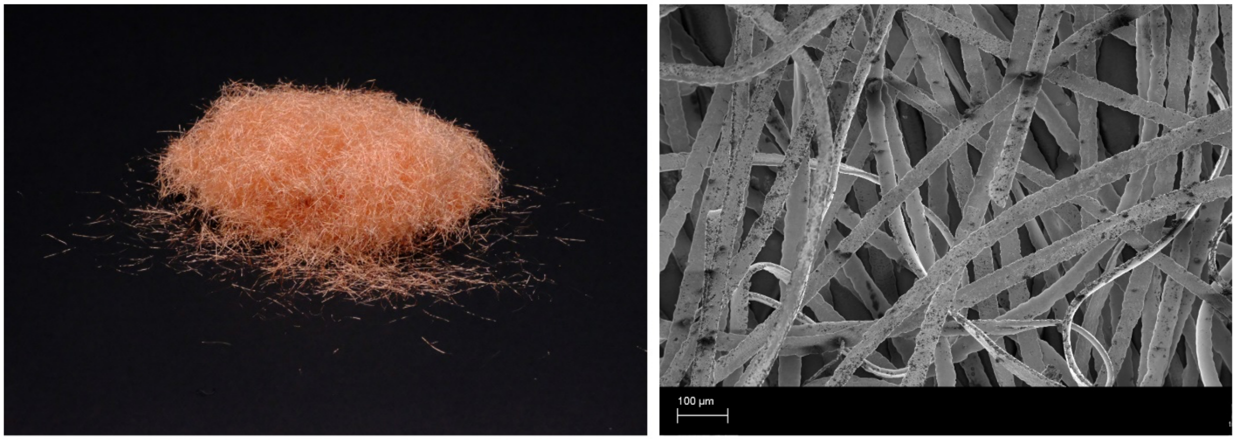

Figure S12: (a) Optical image of melt-spinning cooper fibers; (b) SEM image of melt-spinning cooper fibers.

Figure S13a, b shows the optical and Micro CT reconstruction of the 3D fleece (1mm thick, porosity: 95%). **Figure S13c** shows the SEM of sintered fleece, the ribbon-shape fibers turn to round shape during thermal sintering. The sintered fiber diameter distribution is calculated based on Micro-CT scan of the fleece with software Geodict 2024 (from Math2Market, result is in **Figure S13d**). The diameter of fibers distributed in a narrow range with an average value of 11 μm.

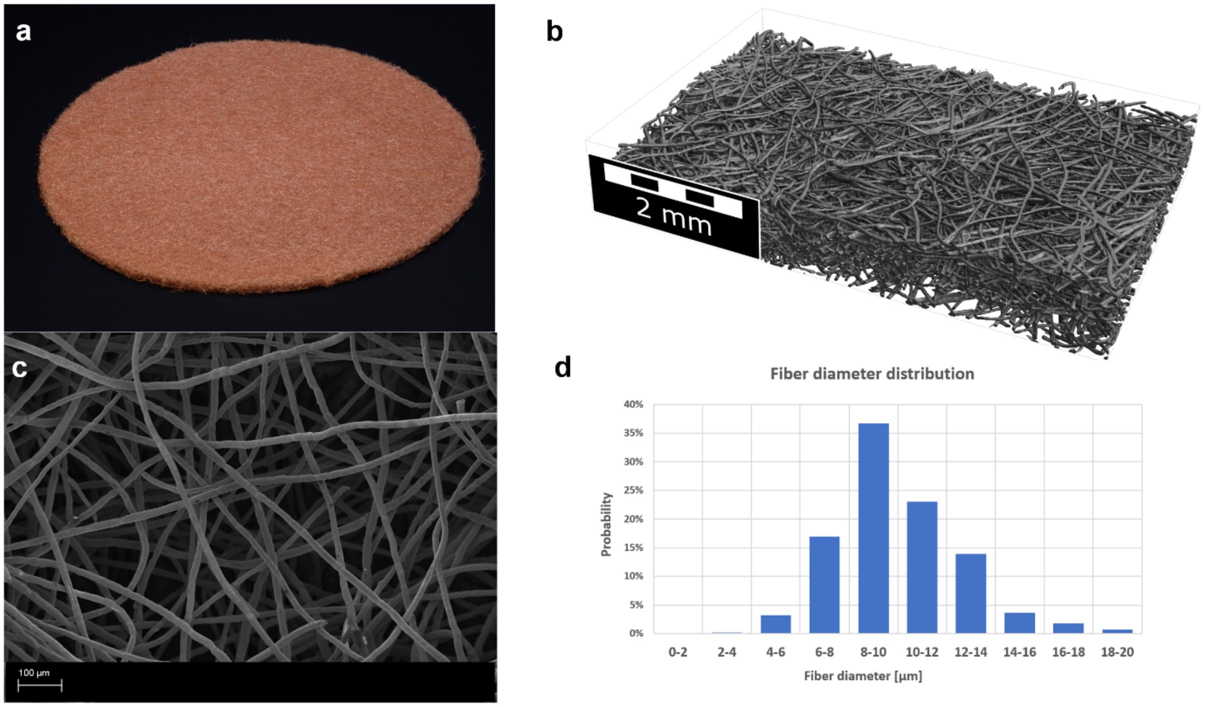

Figure S13: (a) Optical image of the sintered copper fleece; (b) Micro-CT 3D reconstruction of the fine copper fleece; (c) SEM image of the sintered copper fleece; (d) round fiber diameter distribution of the copper fleece.

Figure S14a depicts the pore size distribution of this fleece. The mean pore diameter is approx. 160 μm, the pore size distribution is controlled by fiber density, shape, size and dispersion parameters. Here, we fabricate the fleece with 95% porosity to ensure a high amount of active material filling, and with 160 μm mean pore diameter so that the maximum distance between active material particle and fiber is less than 80 μm. Therefore, the electronic and ionic transport from the fiber to active material is ensured. **Figure S14b** shows the visualized pore size distribution and **Figure S14c** shows the density map of the fleece in XZ-plane. The fiber distribution is rather homogeneous and therefore the pore size distribution is relatively narrow, only some surface pores are larger than 200 μm.

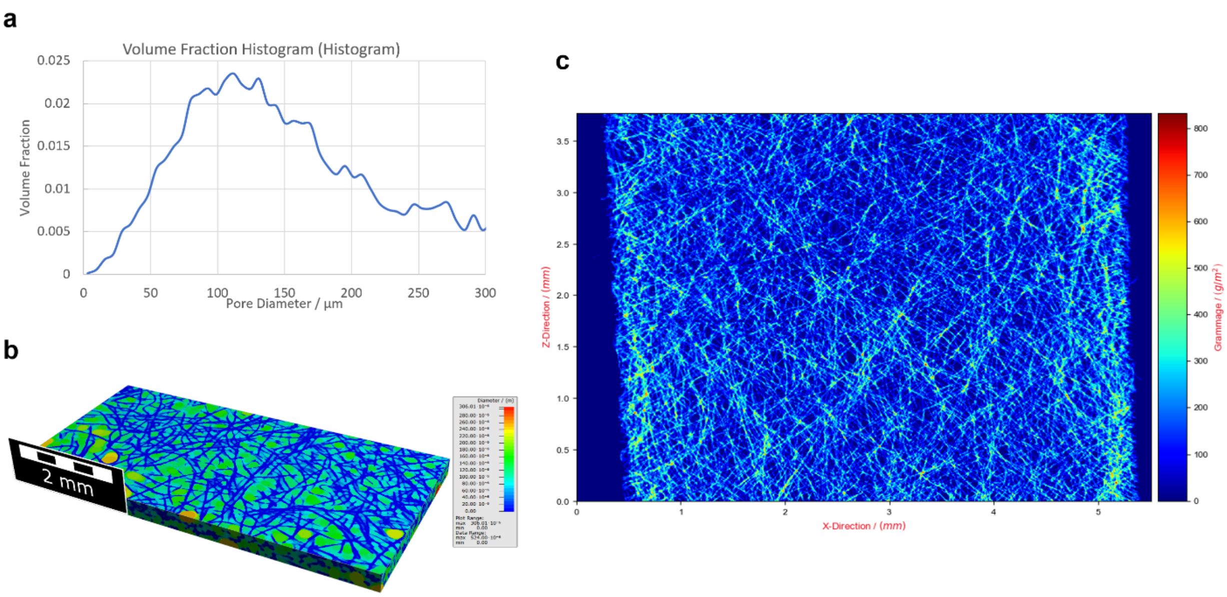

Figure S14: (a) Pore size distribution of copper fleece; (b) visualized pore size distribution of copper fleece; (c) Density map of the copper fleece.

Table S2: Detailed copper fleece parameters:

Fiber Diameter	10~12 µm
Fleece Thickness	400 ~ 2500 µm (before active material filling)
Fleece Porosity	4.8% ~ 5.5%
Fleece Density	442 ~ 450 mg/cm^3
Mean Pore Diameter	148 ~ 167 µm

Electrode characterization

The slurry is then filled into the fleece, and the dry fleece electrode is compressed with a uniaxial hot compression machine to the target active material density. The filling of the 3D fleece requires re-adjustment of slurry viscosity, details are explained in **Method**. The mass load of the active material depends on the fleece thickness and slurry composition. The areal mass load, active material density, electrode thickness, theoretical areal capacity and GITT measured solid phase

diffusivity is shown in **Table S3** and **Table S4**. Worthwhile mentioning that, the active material loading is the areal weight of synthetic graphite (carbon black and binder are not included), the electrode density is the density of the electrode (including the carbon black and binder content). For the fleece electrode, the electrode density is calculated as:

Electrode density = (Electrode mass – Fleece mass) / (Electrode volume – Fleece volume)

The slurry filling quality and electrochemical data including capacity, initial columbic efficiency, rate performance and impedance measurement are shown in **Figure 4.**

Table S3: copper film electrode's parameters.

Foil electrode				
Electrode thickness [um]	Theoretical areal capacity [mAh/cm^2]	Active material load [mg/cm^2]	Electrode density [g/cm^3]	Solid phase diffusivity [cm^2/s]
29.5	0.79	2.26	0.85	1.14×10^{-10}
39.2	1.00	2.86	0.82	8.32×10^{-11}
73.8	1.79	5.12	0.77	6.31×10^{-11}
112.1	2.54	7.27	0.72	4.41×10^{-11}
154.3	3.62	10.36	0.745	3.80×10^{-11}
284	7.16	20.48	0.80	4.87×10^{-12}
457.2	12.10	34.61	0.84	1.38×10^{-12}
50	1.65	4.73	1.05	6.45×10^{-11}
94	3.20	9.15	1.08	3.98×10^{-11}
121	3.51	10.03	0.92	1.62×10^{-11}
150	4.58	13.11	0.97	7.94×10^{-12}

160	5.54	15.86	1.1	4.17×10^{-12}
220	7.62	21.81	1.1	6.76×10^{-13}
80	2.90	8.29	1.15	1.15×10^{-10}
108	4.02	11.49	1.18	8.13×10^{-11}
129	4.92	14.07	1.21	2.57×10^{-11}
200	7.50	21.45	1.19	4.68×10^{-12}
215	8.67	24.80	1.28	3.39×10^{-12}
252	9.37	26.80	1.18	2.57×10^{-12}

Table S4: copper fleece electrode's parameters

Fleece electrode				
Thickness [um]	Theoretical areal capacity [mAh/cm^2]	Active material load [mg/cm^2]	Electrode density [g/cm^3]	Solid phase diffusivity [cm^2/s]
472	8.70	24.85	0.65	1.21×10^{-10}
760	15.52	44.33	0.72	6.92×10^{-11}
870	17.27	49.34	0.7	5.49×10^{-11}
1220	22.83	65.24	0.66	4.07×10^{-11}
1706	33.86	96.75	0.7	3.47×10^{-11}
335	9.50	27.14	1	1.01×10^{-10}
450	12.12	34.64	0.95	1.03×10^{-10}
500	15.60	44.56	1.1	7.59×10^{-11}

1030	27.75	79.28	0.95	3.46×10^{-11}
1278	35.15	100.44	0.97	1.91×10^{-11}
370	13.12	37.45	1.25	7.24×10^{-11}
450	15.44	44.12	1.21	5.89×10^{-11}
550	19.18	54.81	1.23	5.01×10^{-11}
812	26.48	75.66	1.15	3.55×10^{-11}
900	30.63	87.50	1.20	1.95×10^{-11}
1120	37.48	107.08	1.18	1.29×10^{-11}

The areal capacity @ 0.1C, volumetric and gravimetric energy density of foil and fleece electrodes are shown in **Table S5**, here the current collector (CC) mass and volume are also taken into consideration when calculating volumetric and gravimetric energy density.

Table S5: Energy density comparison between foil and fleece electrodes

Foil electrodes					
Electrorde thickness [µm]	Electrode density [g/cm3]	Areal capacity @ 0.1C [mAh/cm2]	Electrode mass including CC [mg/cm2]	Volumetric energy density including CC [mAh/cm3]	Gravimetric energy density including CC [mAh/g]
39.5	0.85	0.785	11.41	158.59	38.65
49.2	0.82	1.08	12.08	182.43	51.48
83.8	0.77	1.72	14.59	183.37	73.23
122.1	0.72	2.41	16.98	182.44	93.13

164.3	0.745	3.42	20.41	196.21	116.68
294	0.8	6.16	31.66	202.63	151.89
467.2	0.84	8.5	47.36	178.12	151.10
60	1.05	1.63	14.16	232.86	70.70
104	1.08	3	19.07	263.16	107.27
131	0.92	3.28	20.04	232.62	113.32
160	0.97	4	23.47	235.29	123.58
170	1.1	4.72	26.52	262.22	133.25
230	1.1	5.62	33.13	234.17	133.70
90	1.15	2.9	18.11	290.00	107.36
118	1.18	3.52	21.67	275.00	115.16
139	1.21	3.92	24.53	263.09	117.25
210	1.19	4.5	32.73	204.55	108.09
225	1.28	5.12	36.46	217.87	112.89
262	1.18	5.07	38.68	186.40	106.56
Fleece electrodes					
Electrorde thickness [µm]	Electrode density [g/cm3]	Areal capacity @ 0.1C [mAh/cm2]	Electrode mass including CC [mg/cm2]	Volumetric energy density including CC [mAh/cm3]	Gravimetric energy density including CC [mAh/g]
472	0.65	8.5	45.85	180.08	185.37
760	0.72	14.52	78.15	191.05	185.80
870	0.7	15.97	88.06	183.56	181.36

1220	0.66	21.83	119.53	178.93	182.63
1706	0.7	29.86	172.67	175.03	172.93
335	1	9.4	42.22	280.60	222.67
450	0.95	11.9	54.89	264.44	216.80
500	1.1	14.1	67.06	282.00	210.26
1030	0.95	25.95	125.63	251.94	206.56
1278	0.97	32.15	157.95	251.56	203.55
370	1.25	12.18	54.47	329.19	223.61
450	1.21	13.94	64.82	309.78	215.06
550	1.23	17.28	80.11	314.18	215.70
812	1.15	24.48	113.01	301.48	216.61
900	1.2	28.43	128.90	315.89	220.56
1120	1.18	31.48	158.60	281.07	198.49

The electrical conductivity of film and fleece electrodes is measured. For characterization of electrical conductivity of the film and fleece electrode, we implemented electrochemical impedance measurement (EIS). By applying equivalent circuit fitting, the ohmic resistance R_s can be calculated by **Equation S12**:

$$R = \frac{l}{\sigma S} \qquad \textbf{Equation S12}$$

Here, σ is the conductivity, l is thickness of the electrode and S is the area of the electrode. Worthwhile mentioning that with EIS, we obtain a SOC dependent electronic conductivity, because during lithiation/de-lithiation the crystal structure of graphite is modified and thus the conductivity of graphite varies. We measured the electronic conductivity of foil electrode and fleece electrode at 150 mV open circuit voltage. **Table S6** shows the result:

Table S6: Electrical DC conductivity of foil and fleece anodes.

Electrode design	AM Density (g/cm^3)	Electrical Conductivity (S/m)
foil	~0,8	350.632 ± 17.81
foil	~1,0	373.120 ± 12.34
foil	~1,2	392.150 ± 20.76
fleece	~0,7	603.1309 ± 40.38
fleece	~0,9	721.4436 ± 67.19
fleece	~1,2	1130.942 ± 80.28

From these data we know that the metal fleece can not only decrease the diffusive resistance but also decrease the electrical resistance. The fleece works as a 3D percolation network for electron transport and reduces the electrical potential gradient within the electrode. Thus, all the graphite particles are at the inter/deintercalation potential of Li into graphite. Then the question comes: the better electrochemical performance in fleece electrodes is because of small electrical resistance or small diffusive resistance? here we carried out a series of simulations based on DFN model to study the influence of electrical conductivity on electrochemical performance of foil electrodes with different thickness. **Table S7** lists the governing equation of the DFN model. Within the electrolyte phase, Nernst-Plank equation and the law of electroneutrality govern the potential and concentration gradient variation. Within the active material, solid-state diffusion and Ohm's law define the lithium concentration and electrical potential level. At the electrochemically active interface, the molar flux and the current flux of each phase are determined by the Butler-Volmer equation.

Table S7: Governing equations of DFN model

Electrolyte (e)	
Diffusion and migration	$\frac{\partial c^e}{\partial t} = \nabla \cdot \vec{N}^e = \cdot \left[\left(D^e + K^e t_+ (1 - t_+) \frac{\mathbb{R}T}{\mathbb{F}^2} \frac{1}{c^e} \right) \nabla c^e + K^e \frac{t_+}{\mathbb{F}} \nabla \varphi^e \right]$
Charge conservation and electroneutrality	$0 = \nabla \cdot \vec{j}^e = \nabla \cdot \left[\left(K^e (1 - t_+) \frac{\mathbb{R}T}{\mathbb{F}} \frac{1}{c^e} \right) \nabla c^e + K^e \nabla \varphi^e \right]$
**Active material (s)**	
Diffusion	$\frac{\partial c^s}{\partial t} = \nabla \cdot \vec{N}^s = \nabla \cdot [D^s \nabla c^s]$
Ohm's law	$0 = \nabla \cdot \vec{j}^s = \nabla \cdot [K^s \nabla \Phi^s]$
**Interface between electrolyte and active material**	
Butler Volmer current density	$i_{se}(c^e, c^s, \varphi^e, \Phi^s) = k \sqrt{c^s c^e \left(1 - \frac{c^s}{c^s_{max}}\right)} 2 sinh \left[\frac{\mathbb{F}}{2\mathbb{R}T} \left(\underbrace{\Phi^s - \varphi^e - U_0}_{=\eta_s} \right) \right]$

Variables, parameters and constants

c^e:	Li-ion concentration in e
c^s:	Li concentration in s
φ^e:	Electrochemical potential of e
Φ^s:	Electrical potential of s
$\vec{N}^e$:	Molar flux of Li-ion in e
$\vec{N}^s$:	Diffusive flux of Li in s
$\vec{j}^e$:	Current density of all ionic charges in e
$\vec{j}^s$:	Effective electronic current in s
T:	Temperature
U_0:	Open circuit voltage of active material vs. Lithium
η_s:	Interfacial overpotential
c^s_{max}:	Maximum Li concentration in s
K^e:	Ionic conductivity of e
K^s:	Electronic conductivity of s
D^e:	Ionic diffusion constant of e
D^s:	Diffusion constant of s
t_+:	Transference number
k:	Butler-Volmer rate constant
$\mathbb{F}$:	Faraday constant
$\mathbb{R}$:	Universal gas constant

Figure S15 shows the simulation results of anode half cells. Increasing the graphite anode electrical conductivity did not improve electrode performance. Besides, despite very good electrical conductivity, charging and discharging at 1C was impossible at electrode thicknesses above ~120 µm. At 160 µm anode thickness only ~35% percent of theoretical capacity could be

reached at 1C. These calculations demonstrate quite well that at least in anodes, the bottleneck of thick electrode electrochemical performance is not electrical conductivity. Also, the simulation indicates that there must be another mechanism existing in fleece electrodes that helps the fleece electrodes to work properly.

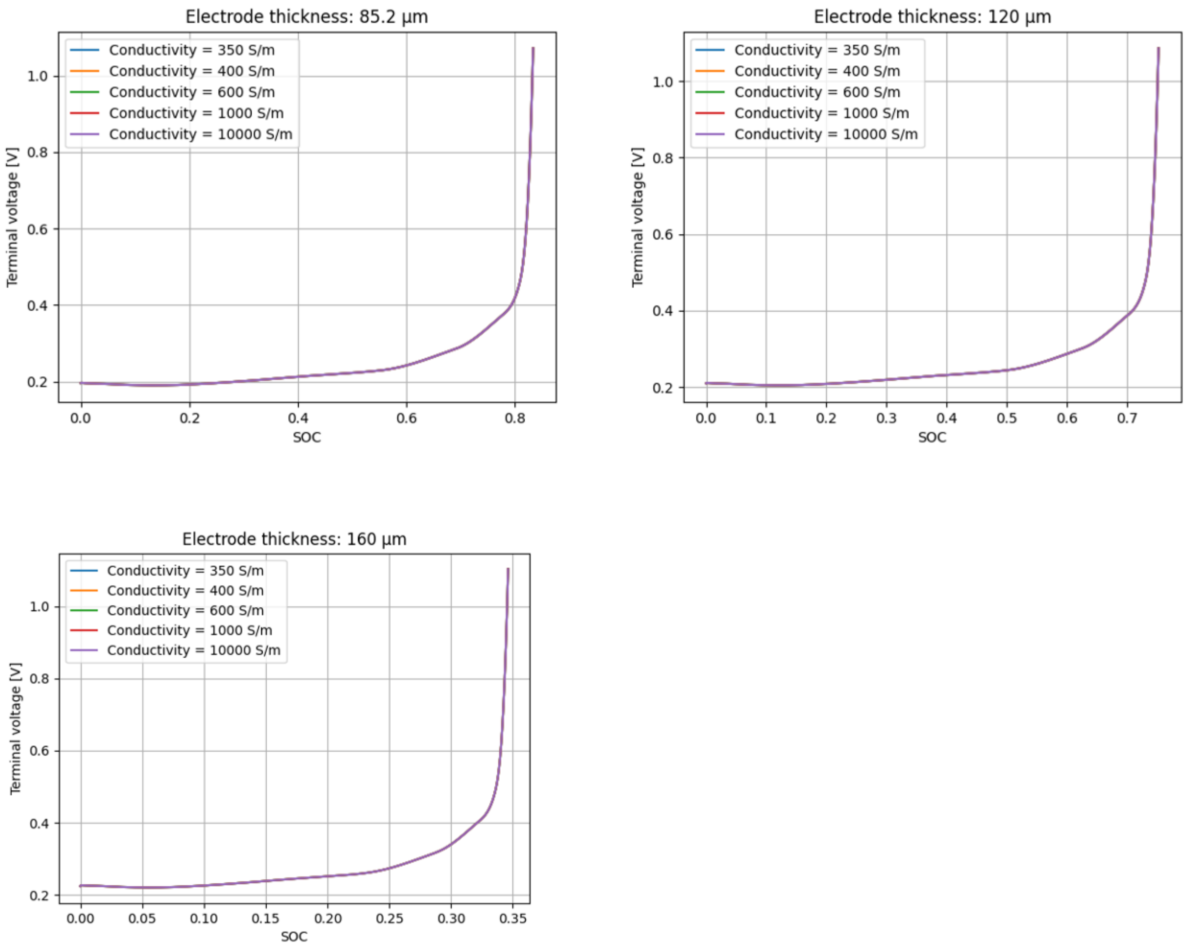

Figure S15: Half-cell anode DFN simulation of electrodes with different electrical conductivity and thickness.

Supporting Information V: GITT and μ-CT reconstruction

The galvanostatic intermittent titration technique (GITT) is a method that provides kinetic and thermodynamic information about an electrochemical system. We used GITT to investigate the diffusion process within the anode half-cell. During the measurement a series of current pulses (C/20), each followed by a relaxation time of 2h, is applied to the anode half-cell. During the relaxation phase the open circuit voltage (OCV) can be determined as a function of the state of charge (SoC). We chose 100 pulses during both charging and discharging to obtain a more accurate OCV and more detailed information concerning diffusion process variations at different SoCs. The relaxation periods were chosen to be sufficiently long to ensure the establishment of equilibrium conditions at the lithium-ion concentration gradient within the electrolyte. **Figure S16a** shows the GITT curve. The OCVs for lithiation and delithiation can be extracted by plotting the relaxation-phase voltage. By comparing the pulse phase and relaxation phase, the solid-state diffusivity was calculated utilizing **Equation S12**[4]. Here, I is the charging current during the pulse phase, V_m is the molar volume of the active material, F is the Faraday constant, S is the active material/electrolyte interface area, and y, which ranges from 0 to 1, indicates the SOC. The interfacial area between the active material and the electrolyte was calculated with the aid of a micro-CT reconstructed 3D structure. **Figure S16b** describes the solid-state diffusivity as a function of the OCV. Two gaps, one at approximately 90 mV and one at approximately 140 mV, were observed, because intercalation limits the diffusion rate according to E. Deiss. Here we employ the mean value of the diffusivity at the plateau phase at around 180 mV.

$$D = \frac{4}{\pi}\left(\frac{IV_m}{FS}\right)^2\left(\frac{dE_s/dy}{dE_t/dt^{\frac{1}{2}}}\right)^2$$ **Equation S12**

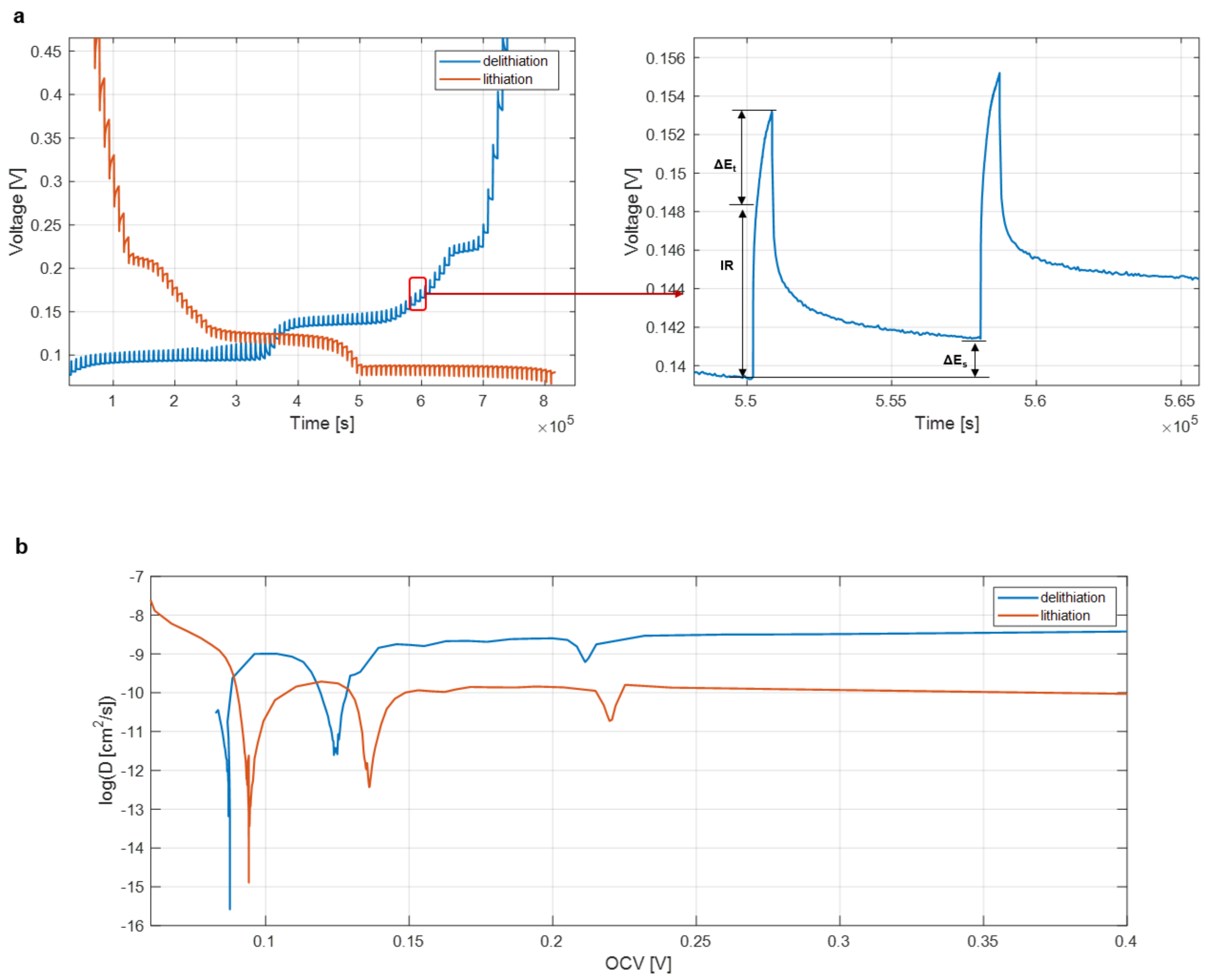

Figure S16: (a) GITT data obtained from a half-cell anode; (b) The solid-phase lithium-ion diffusivity as calculated by GITT.

Figure S17a shows the diffusion coefficient plotted against the OCV in cells of varying thickness, all containing the metallic fleece. **Figure S17b** shows the diffusion coefficient plotted against the OCV in conventional cells of varying thickness (none of them containing the metallic fleece). To facilitate a comparison of the solid-phase diffusion coefficient between fleece-containing and conventional batteries, we focused on the section where the graph plateaus at OCV = 0.2V. The results are shown in **Figure 2**.

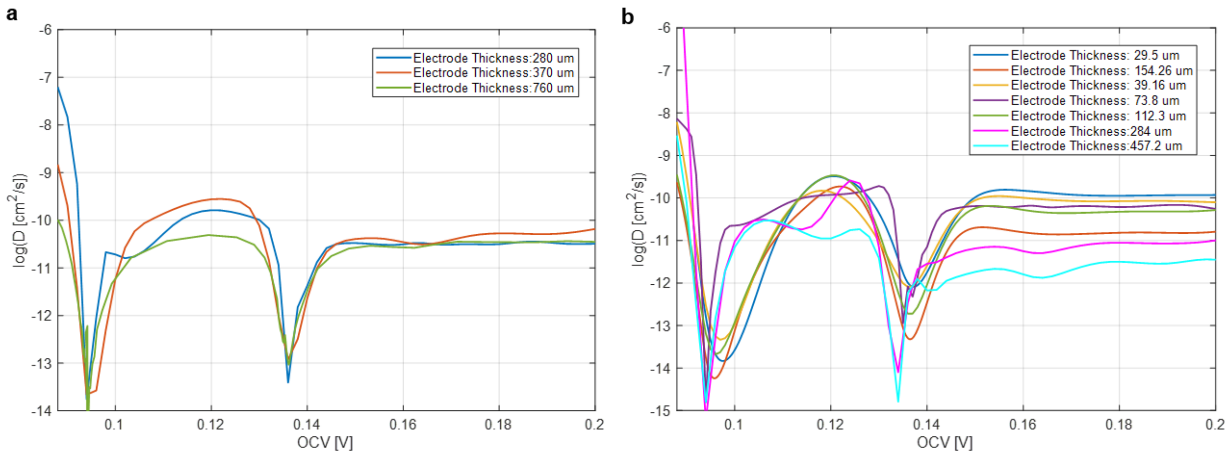

Figure S17: (a) The diffusion coefficient plotted against the OCV of fleece batteries with varying electrode thickness; (b) the diffusion coefficient plotted against the OCV of conventional batteries with varying electrode thickness.

Unfortunately, GITT is unreliable when it comes to measuring the chemical diffusivity at the electrode/electrolyte interface. Unlike cathode materials like NMC or LFP, which have a bimodal particle size distribution, the graphite particle is ‘smoother’. **Figures S18a** and **S18b** show the battery-grade graphite particle, which is derived from natural graphite flakes with differing crystalline orientation (**Figure S18c**). Because the particle shape cannot be approximated as round or elliptical, we utilized μ-CT as the preferred method for identifying particle size, particle size distribution and shape, as it allows greater resolution and accuracy than BET or estimation by particle size distribution.

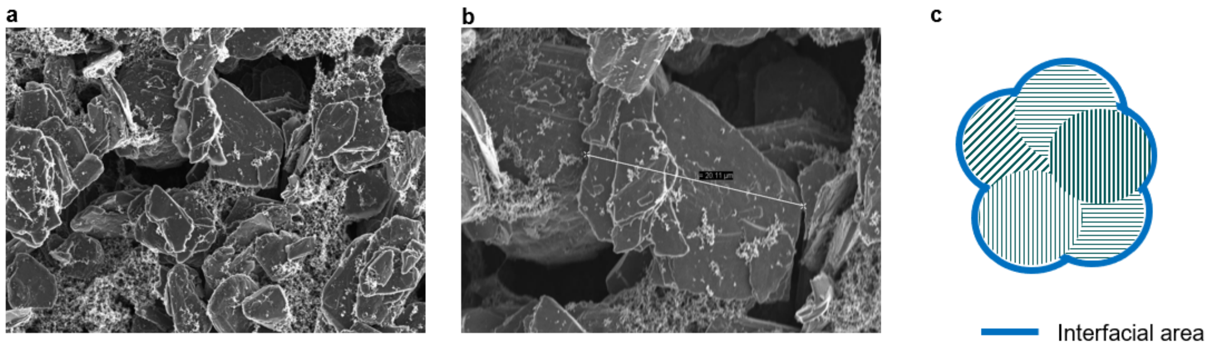

Figure S18: (a) and (b) SEM images of battery grade-graphite particles; (c) Illustration of the fused graphite particle with different crystalline directions.

Figure S19a shows a conventional lithium-ion battery anode scanned and reconstructed by X-ray μ-CT. The 3D model captures microstructural details of the graphite particles and CBP. Due to the similar attenuation coefficients of the two phases, the segmentation of the two phases is challenging. To better reconstruct and distinguish details, a grain fitting algorithm was implemented to separate the graphite particles from the electrode, and the rest phase was assigned as CBP (**Figure S19b**). Utilizing the 3D reconstructed data, the interfacial area of each cell was calculated.

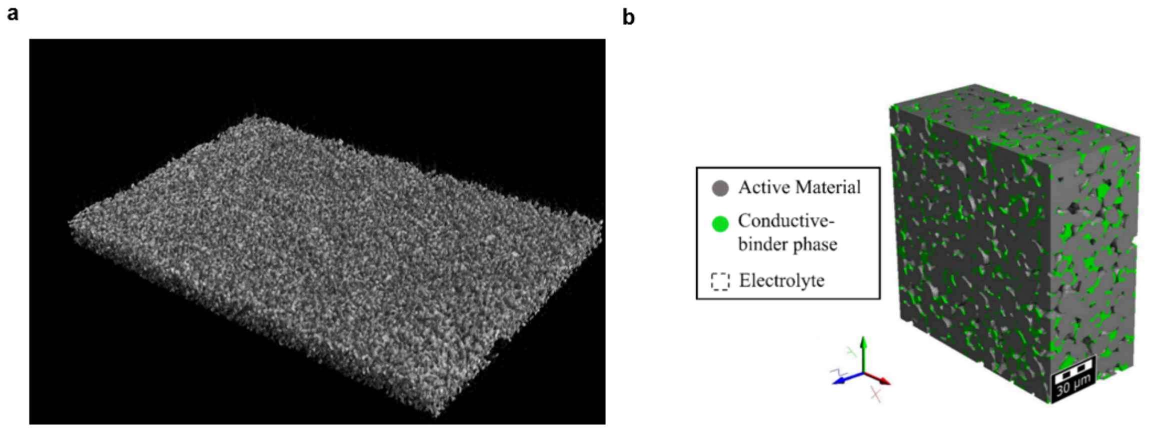

Figure S19: (a) μ-CT 3D reconstruction of the lithium-ion battery anode; (b) segmentation of the 3D reconstructed structure.

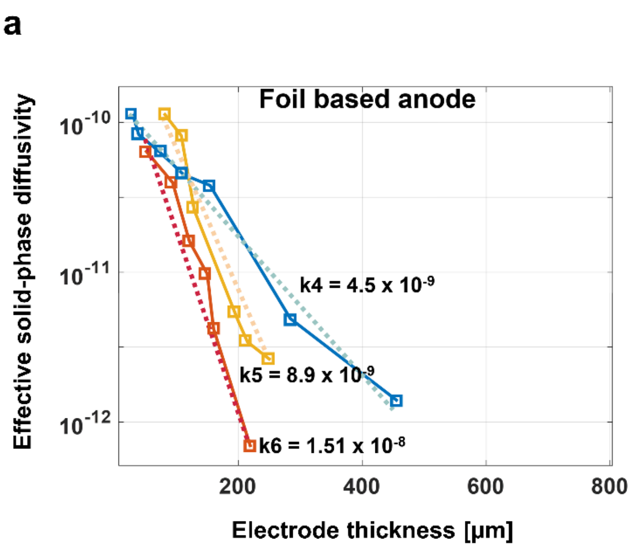

Figure S20: (a) Effective solid-phase diffusivity of foil anodes of different thickness and density measured by GITT, and its linear fitting; (b) Effective solid-phase diffusivity of fleece anodes of different thickness and density measured by GITT, and its linear fitting.

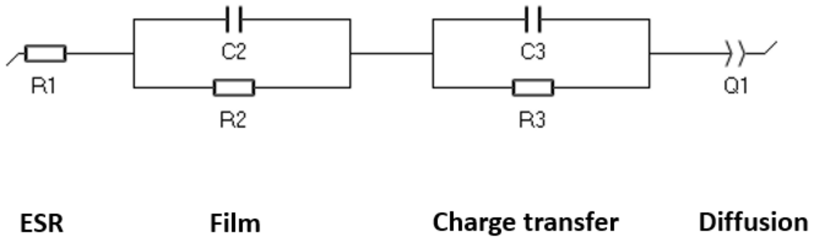

Figure S21: Equivalent circuit for EIS data fitting.

Figure S21 shows the equivalent circuit for EIS data fitting. Here R1 represent the total equivalent series resistance (ESR), film impedance and charge transfer impedance is represented by a parallelly connected capacitor and resistor. The Q1 is constant-phase element (CPE), which represent the diffusion process.

Table S7: EIS fitting parameters of foil and fleece electrodes

Fitting parameter	Foil electrode (86 µm)	Fleece electrode (276 µm)
R1 [mOhm.m^2]	0.3817	0.3862
R2 [mOhm.m^2]	1.2475	0.4057
C2 [F.m^2]	0.1719e-6	0.3121e-7
R3 [mOhm.m^2]	3.0272	1.6236
C3 [F.m^2]	6.905e-6	8.021e-6
Q1 [F.s$^{(a-1)}$]	0.09112	0.07933
a	0.8319	0.6948

REFERENCES

1. Peters, C. *et al.* Multicomponent diffusion coefficients from microfluidics using Raman microspectroscopy. *Lab Chip* **17**, 2768–2776 (2017).

2. Peters, C., Thien, J., Wolff, L., Koß, H.-J. & Bardow, A. Quaternary Diffusion Coefficients in Liquids from Microfluidics and Raman Microspectroscopy: Cyclohexane + Toluene + Acetone + Methanol. *J. Chem. Eng. Data* **65**, 1273–1288 (2020).

3. Oldham, K. B. A Gouy–Chapman–Stern model of the double layer at a (metal)/(ionic liquid) interface. *Journal of Electroanalytical Chemistry* **613**, 131–138 (2008).

4. Verma, A. *et al.* Galvanostatic Intermittent Titration and Performance Based Analysis of LiNi0.5Co0.2Mn0.3O2 Cathode. *J. Electrochem. Soc.* **164**, A3380 (2017).
